# VIRMA promotes nasopharyngeal carcinoma, tumorigenesis, and metastasis by upregulation of *E2F7* in an m6A-dependent manner

**DOI:** 10.1016/j.jbc.2023.104677

**Published:** 2023-04-05

**Authors:** Zi-Qi Zheng, Zhuo-Hui Huang, Ye-Lin Liang, Wei-Hong Zheng, Cheng Xu, Zhi-Xuan Li, Na Liu, Pan-Yang Yang, Ying-Qin Li, Jun Ma, Ying Sun, Ling-Long Tang, Denghui Wei

**Affiliations:** State Key Laboratory of Oncology in South China, Guangdong Key Laboratory of Nasopharyngeal Carcinoma Diagnosis and Therapy, Collaborative Innovation Center of Cancer Medicine, Sun Yat-sen University Cancer Center, Guangzhou, China

**Keywords:** nasopharyngeal carcinoma, metastasis, m6A, VIRMA, E2F7

## Abstract

The N6-methyladenosine (m6A) modification possesses new and essential roles in tumor initiation and progression by regulating mRNA biology. However, the role of aberrant m6A regulation in nasopharyngeal carcinoma (NPC) remains unclear. Here, through comprehensive analyses of NPC cohorts from the GEO database and our internal cohort, we identified that VIRMA, an m6A writer, is significantly upregulated in NPC and plays an essential role in tumorigenesis and metastasis of NPC, both *in vitro* and *in vivo*. High VIRMA expression served as a prognostic biomarker and was associated with poor outcomes in patients with NPC. Mechanistically, VIRMA mediated the m6A methylation of E2F7 3′-UTR, then IGF2BP2 bound, and maintained the stability of E2F7 mRNA. An integrative high-throughput sequencing approach revealed that E2F7 drives a unique transcriptome distinct from the classical E2F family in NPC, which functioned as an oncogenic transcriptional activator. E2F7 cooperated with CBFB-recruited RUNX1 in a non-canonical manner to transactivate ITGA2, ITGA5, and NTRK1, strengthening Akt signaling-induced tumor-promoting effect.

Nasopharyngeal carcinoma (NPC), a clinically occult and lethal head and neck cancer, is particularly prevalent in East and Southeast Asia (1). In the last 3 decades, the survival of patients with early-stage NPC has dramatically improved with the development of combined therapy strategies, and the 5-year local–regional control rate has exceeded 90%. However, most newly diagnosed patients are already in the advanced stage and are prone to relapse or metastasis even after current standard treatment (2, 3, 4). Currently, distant metastasis has become the leading cause of death in patients with NPC (2). Therefore, determining the mechanisms underlying NPC progression and metastasis will lead to the identification of tumor vulnerabilities and the development of novel therapeutic strategies.

Emerging evidence shows that epigenetic dysregulation frequently leads to malignant diseases, underscoring the importance of alterations not involving DNA sequences in tumor cells (5). Analogous to the well-characterized chemical modifications on DNA or histones, the N6-methyladenosine (m6A) modification of RNA, the most prevalent internal chemical modification in polyadenylated RNA, represents a crucial layer of posttranscriptional regulation of gene expression (6). m6A precisely regulates the transcription, processing, splicing, localization, and degradation of mRNAs or non-coding RNAs through a variety of m6A regulators. The methyltransferase complex, comprising methyltransferase-like 3 (METTL3) and METTL14 as the catalytic core, and several protein factors, such as vir-like m6A methyltransferase associated (VIRMA, also known as KIAA1429) and Wilms tumor 1-associated protein (WTAP), are responsible for the deposition of m6A modification in RNAs. By contrast, two Fe(II)/2-oxoglutarate-dependent dioxygenases, AlkB homolog 5 (ALKBH5) and FTO alpha-Ketoglutarate dependent dioxygenase (FTO), selectively remove the methyladenosine through an oxidative demethylation mechanism. The methyladenosine moiety is recognized by m6A-binding proteins, such as insulin-like growth factor 2 mRNA-binding proteins (IGF2BPs) and YTH domain-containing proteins (YTHDFs), which play multifunctional roles in RNA fate determination (7, 8, 9).

Recently, alteration of m6A regulators and subsequent dysregulation of global m6A abundance have been reported to influence cancer hallmarks (10, 11). For example, METTL3 and METTL14 control hematopoietic stem and progenitor cell differentiation and sustain proliferative signaling by promoting mRNA stability and translation of MYC (12, 13). *YTHDF2* silencing provoked tumor-promoting inflammation, vascular reconstruction, and energy metabolism reprogramming in hepatocellular carcinoma by inhibiting the degradation of m6A-modified *IL11* (interleukin 11) and *SERPINE2* (serpin family E member 2) transcripts (14). *FTO* is a potential target in leukemia, the depletion of which dramatically impaired leukemia stem cell, initiated cell-renewal ability, and suppressed immune evasion (15). Our previous work has shown that the m6A modification might participate in NPC development. Oncogenic long non-coding RNA *FAM225A*, upregulated by m6A modification, acts as competing endogenous to sponge miR-590-3p/miR-1275, thereby promoting NPC development (16). Moreover, m6A-mediated *ZNF750* (zinc finger protein 750) repression inhibits NPC cell apoptosis and modulates NPC progression (17). These studies revealed an underlying disordered m6A regulatory network in NPC. However, the roles of m6A regulators and sequential m6A dysregulation in NPC remain largely unknown.

In the present study, we investigated the role of VIRMA and its underlying mechanisms in NPC. We found that *VIRMA* is upregulated in NPC and promotes NPC cell proliferation and metastasis *in vitro* and *in vivo* by upregulating *E2F7* (encoding E2F transcription factor 7) expression *via* an m6A-dependent mechanism. VIRMA-mediated *E2F7* m6A modification is recognized by IGF2BP2, which enhances the stability of *E2F7* mRNA. E2F7 cooperates with transcriptional activator RUNX family transcription factor 1 (RUNX1) that is recruited by core-binding factor subunit beta (CBFB) to transactivate integrin subunit alpha (*ITGA2*), *ITGA5*, and neurotrophic receptor tyrosine kinase 1 (*NTRK1*), which consequently promotes NPC progression. These results reveal the critical role of VIRMA that modulates *E2F7* expression to control the transcription program of NPC and provide a potential biomarker and therapeutic target for NPC.

## Results

### *VIRMA* is upregulated in NPC and is associated with the adverse prognosis of patients with NPC

**Figure 1:**
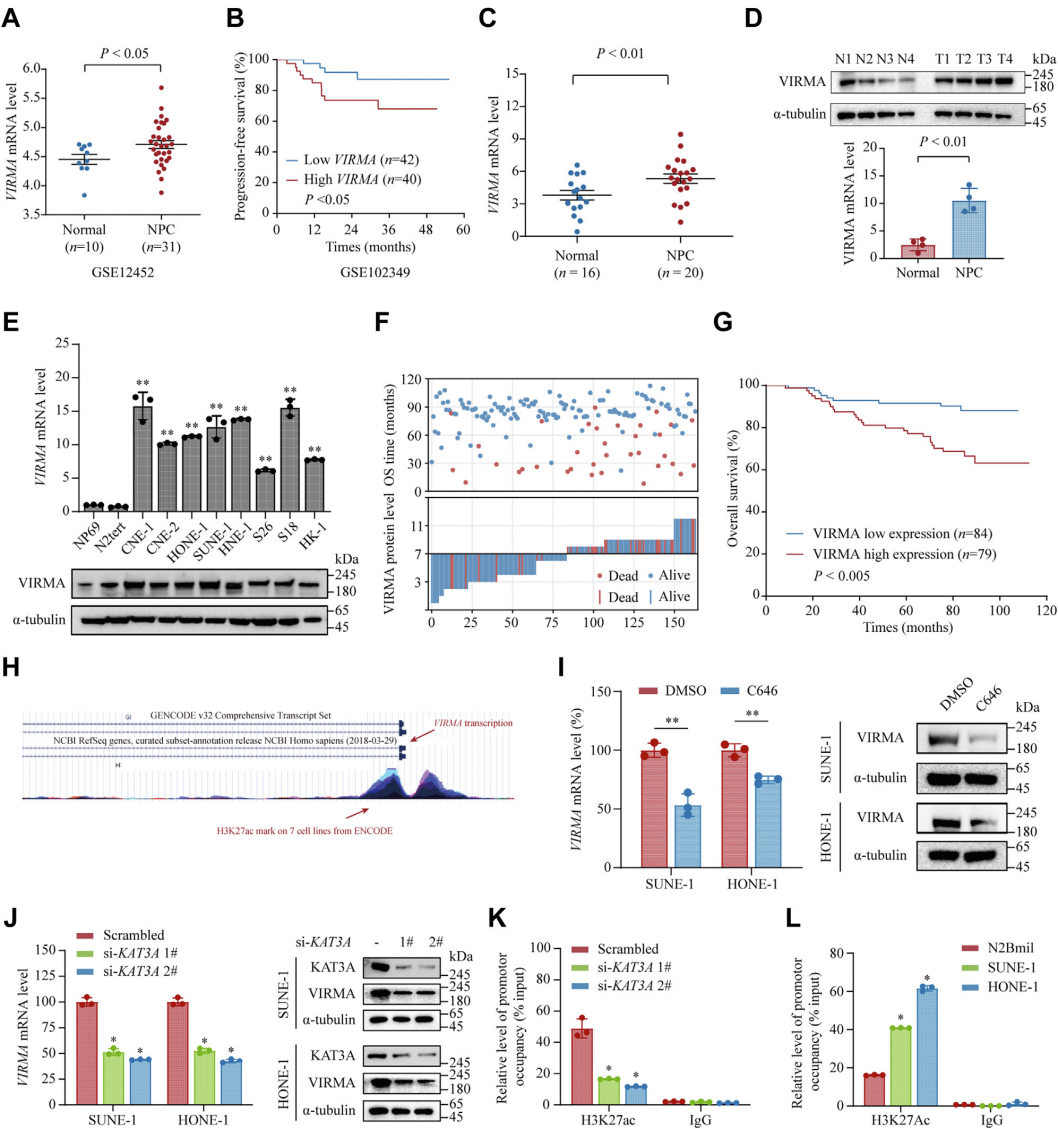
**Upregulation of *VIRMA* in NPC tissues and cell lines is controlled by H3K27 acetylation.***A*, expression levels of *VIRMA* in NPC tissues and normal nasopharyngeal tissues were compared based on the GEO database GSE12452. *B*, Kaplan–Meier analysis was applied to compare the survival between patients with NPC with *VIRMA* low and high expression based on GEO database GSE102349. *C* and *D*, RNA (qPCR analysis) (*C*) and protein (Western blotting analysis) (*D*) levels of *VIRMA* in NPC tissues and normal nasopharyngeal tissues were compared. *E*, RNA (qPCR analysis) and protein (Western blotting analysis) levels of *VIRMA* in NPC cell lines (CNE-1, CNE-2, HONE-1, SUNE-1, HNE-1, S26, S18, and HK-1) and immortalized nasopharyngeal epithelia (NP69 and N2tert). *F*, the overall survival time (*upper panel*) and VIRMA expression (*lower panel*) of our NPC cohort (n = 163). *G*, Kaplan–Meier analysis was applied to compare the survival between patients with VIRMA low and high expression in our NPC cohort. *H*, H3K27 acetylation of the VIRMA promoter region was analyzed based on UCSC Genome Browser (http://genome.ucsc.edu/). The layered H3K27ac tracks show where modification of histone proteins is suggestive of enhancer. Different colors represent the H3K27ac mark (often found near regulatory elements) on 7 cell lines from ENCODE database (https://www.encodeproject.org). *I*, relative expression of *VIRMA* was examined by qPCR (*left panel*) and Western blotting (*right panel*) with or without C646 (10 μM) treatment for 48 h in HONE-1 and SUNE-1 cells. *J*, relative expression of *VIRMA* was examined by qPCR (*left panel*) and Western blotting (*right panel*), with or without *KAT3A* knockdown in NPC cells. *K*, ChIP analysis of H3K27ac enrichment in the *VIRMA* promoter region upon *KAT3A* knockdown. *L*, ChIP analysis of H3K27ac enrichment in *VIRMA* in SUNE-1, HONE-1, and N2Bmil cells. Data are presented as the mean ± SD. ∗*p* < 0.05, ∗∗*p* < 0.01. ChIP, chromatin immunoprecipitation; NPC, nasopharyngeal carcinoma; VIRMA, vir-like m6A methyltransferase associated.

The m6A regulators are frequently altered in different types of cancer (18). To investigate if they were also aberrantly expressed in NPC, we compared their mRNA levels between 31 NPC and 10 nasopharyngeal epithelial tissues from the GEO database (GSE12452). Several m6A regulators, including *WTAP*, *VIRMA*, and *IGF2BP3*, were markedly elevated in NPC tissues. Among them, *VIRMA* was consistently upregulated in NPC tissues and predicted poor prognosis in patients with NPC according to GSE61218 and GSE102349, indicating its crucial role in NPC (Figs. 1, *A* and *B* and S1*A*, all *p* < 0.05). To confirm these findings, we first determined the mRNA and protein levels of *VIRMA* in NPC tissues and normal nasopharyngeal tissues using quantitative real-time reverse transcription PCR (qRT-PCR) and Western blotting, which showed consistent results (Fig. 1, *C* and *D*). Moreover, both the mRNA and protein levels of *VIRMA* were significantly upregulated in NPC cell lines compared with those in immortalized nasopharyngeal epithelial NP69 and N2tert cells (Fig. 1*E*). In addition, we also noticed that *VIRMA* was upregulated in different types of cancer based on The Cancer Genome Atlas data (Fig. S1*B*). Collectively, these results showed that *VIRMA* is upregulated in NPC.

Next, we sought to determine the clinical significance of *VIRMA* in NPC. The expression level of VIRMA was examined using immunohistochemistry (IHC) staining and subsequent immunoreactive score (IRS) analysis in an NPC cohort (n = 163). Based on the median value of the VIRMA score, patients with NPC were stratified into two groups (VIRMA low expression, n = 84; VIRMA high expression, n = 79) as illustrated in Fig. S1*C*. Notably, we found that high VIRMA expression was associated with increased rates of death and distant metastasis (Fig. 1*F* and Table S1). Kaplan–Meier analysis showed that patients with NPC having higher VIRMA expression were associated with shorter overall survival (Fig. 1*G*). To further investigate the prognostic value of *VIRMA*, we performed univariate and multivariate Cox regression analysis. The results showed that VIRMA was an independent prognostic factor for the overall survival of patients with NPC (Fig. S1*D*). To assess the predictive value of VIRMA in different follow-up periods, we performed time-dependent receiver–operating characteristics curve analysis based on the tumor node metastasis (TNM) risk score, VIRMA risk score, and the combination of TNM and VIRMA risk scores. The results showed that the combination of TNM and VIRMA had a relatively stronger predictive ability than TNM alone, which reached the highest prediction efficiency after 4 years (Fig. S1*E*). These findings suggested that *VIRMA* is a promising prognostic biomarker for patients with NPC.

Epigenetic modification plays important role in regulating gene expression (19). To gain an insight into the mechanisms contributing to the upregulation of *VIRMA* in NPC, we analyzed epigenetic modification of the *VIRMA* promoter using the online tool UCSC Genome Browse (http://generic.ucsc.edu/). In the *VIRMA* promoter region, we observed enriched H3 lysine 27 acetylation (H3K27ac), which is a histone modification mark of active genes, across seven cell lines, suggesting that histone acetylation might regulate *VIRMA* expression (Fig. 1*H*). To test this hypothesis, we treated SUNE-1 and HONE-1 with the histone acetyltransferase (HAT) inhibitor C646 and found that inhibition of HAT significantly reduced the mRNA and protein levels of *VIRMA* (Fig. 1*I*, both *p* < 0.05). Since KAT3A and KAT3B are two histone acetyltransferases that are responsible for histone H3K27ac modification (20), we analyzed the expression of *VIRMA* in cells transfected with si-NC (scrambled control), si-*KAT3A*, or si-*KAT3B*. Quantitative RT-PCR and Western blotting showed that the knockdown of *KAT3B* had no impact on *VIRMA* expression, while the knockdown of *KAT3A* apparently decreased expression of *VIRMA* in both RNA and protein levels (Figs. 1*J* and S2, *A* and *B*). Furthermore, we performed ChIP assays and found that the knockdown of *KAT3A* decreased the H3K27ac deposition at the *VIRMA* promoter significantly (Fig. 1*K*). To investigate whether H3K27ac modification contributes to *VIRMA* upregulation in NPC, we compared the H3K27ac modification levels of the *VIRMA* promoter between NPC cell lines (SUNE-1 and HONE-1) and immortalized nasopharyngeal epithelial N2Bmil cells. As expected, H3K27ac modification levels were two- to fourfold higher in NPC cell lines than those in N2Bmil cells (Fig. 1*L*). These results suggested that the elevation of *VIRMA* in NPC could be partially attributed to KAT3A-mediated H3K27ac modification of its promoter.

### VIRMA promotes NPC proliferation, migration, and invasion *in vitro* and *in vivo*

**Figure 2:**
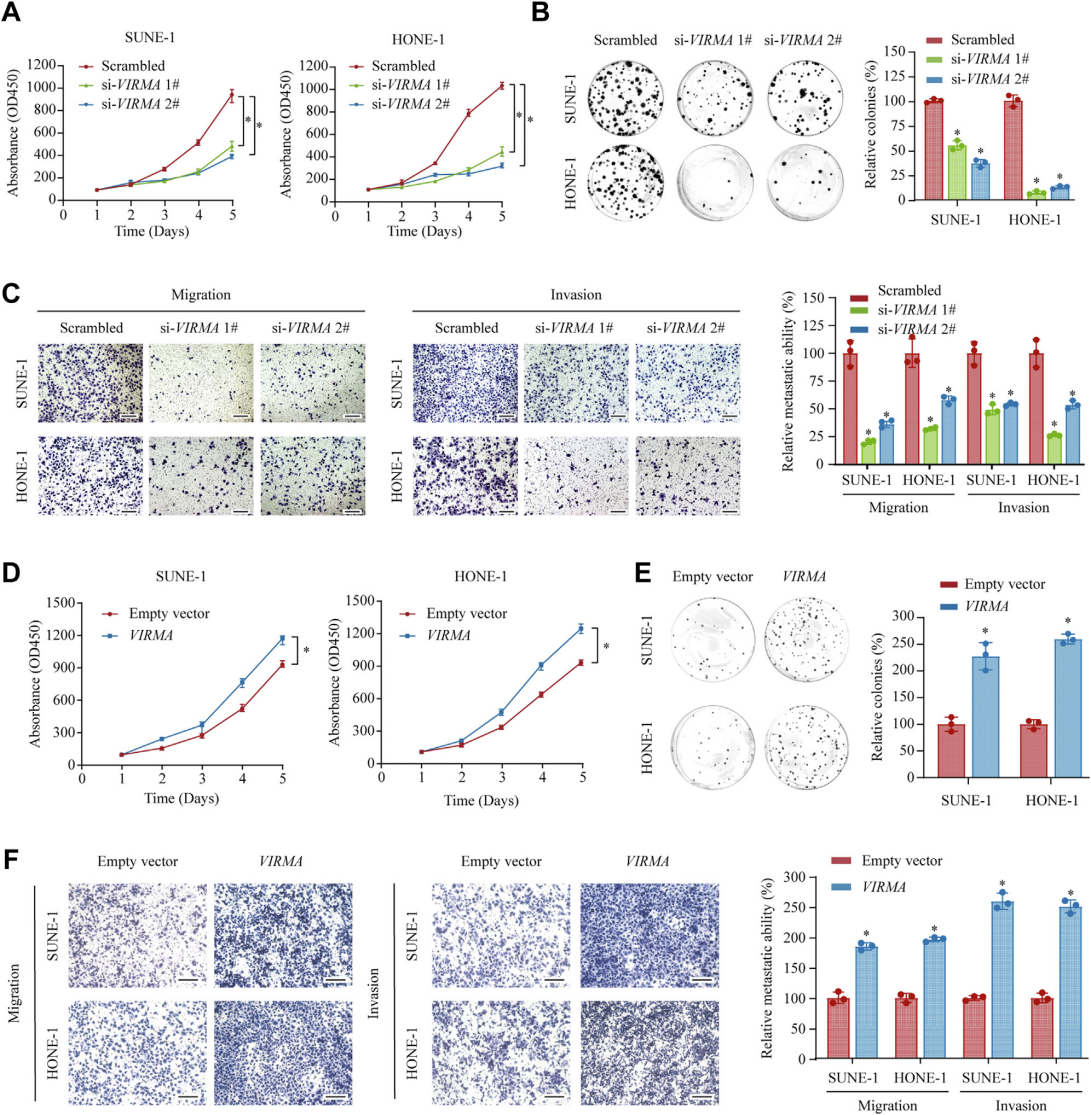
**VIRMA promotes NPC cell proliferation, migration, and invasion *in vitro*.***A*, CCK-8 assays were performed to analyze the proliferation ability of SUNE-1 and HONE-1 cells after silencing of *VIRMA*. Data are presented as the mean ± range. *B*, colony formation assays were conducted to analyze the growth ability of SUNE-1 and HONE-1 cells upon *VIRMA* silencing. *C*, the migration and invasion capacities of *VIRMA*-silenced SUNE-1 and HONE-1 cells were analyzed using Transwell assays. Scale bar: 200 μm. *D*, CCK-8 assays were performed to analyze the proliferation ability of SUNE-1 and HONE-1 cells overexpressing *VIRMA*. Data are presented as the mean ± range. *E*, colony formation assays were conducted to analyze the growth ability of SUNE-1 and HONE-1 cells with *VIRMA* overexpression. *F*, migration and invasion capacities of *VIRMA*-overexpressing SUNE-1 and HONE-1 cells were analyzed using Transwell assays. Scale bar: 200 μm. Data are presented as the mean ± SD. ∗*p* < 0.05. CCK-8, Cell Counting Kit-8; NPC, nasopharyngeal carcinoma; VIRMA, vir-like m6A methyltransferase associated.

To investigate the potential roles of VIRMA in NPC, we first performed gene set enrichment analysis of transcriptomic data from the GEO database (GSE12452). The *VIRMA* expression level correlated positively with NPC development. In addition, we found that high *VIRMA* expression was associated with enhanced NPC proliferation and metastasis ability (Fig. S3*A*), suggesting that VIRMA was a crucial factor promoting NPC development and metastasis. To further validate these findings, we knocked down *VIRMA* in SUNE-1 and HONE-1 cells using two individual siRNAs (si-VIRMA 1# and 2#, Fig. S3*B*). In accordance with previous findings, Cell Counting Kit-8 (CCK-8) and colony formation assays showed that silencing of *VIRMA* significantly impaired the growth capacity of NPC cells (Fig. 2, *A* and *B*). Transwell migration and invasion assays showed that the migration and invasion abilities of NPC cells were significantly decreased upon *VIRMA* knockdown (Fig. 2*C*). Accordingly, overexpression of *VIRMA* markedly increased the proliferation and metastasis capacities of the cells *in vitro* (Figs. S3*C* and 2, *D*–*F*). Collectively, these findings suggested that the upregulation of *VIRMA* contributes to NPC cell growth and metastasis *in vitro*.

**Figure 3:**
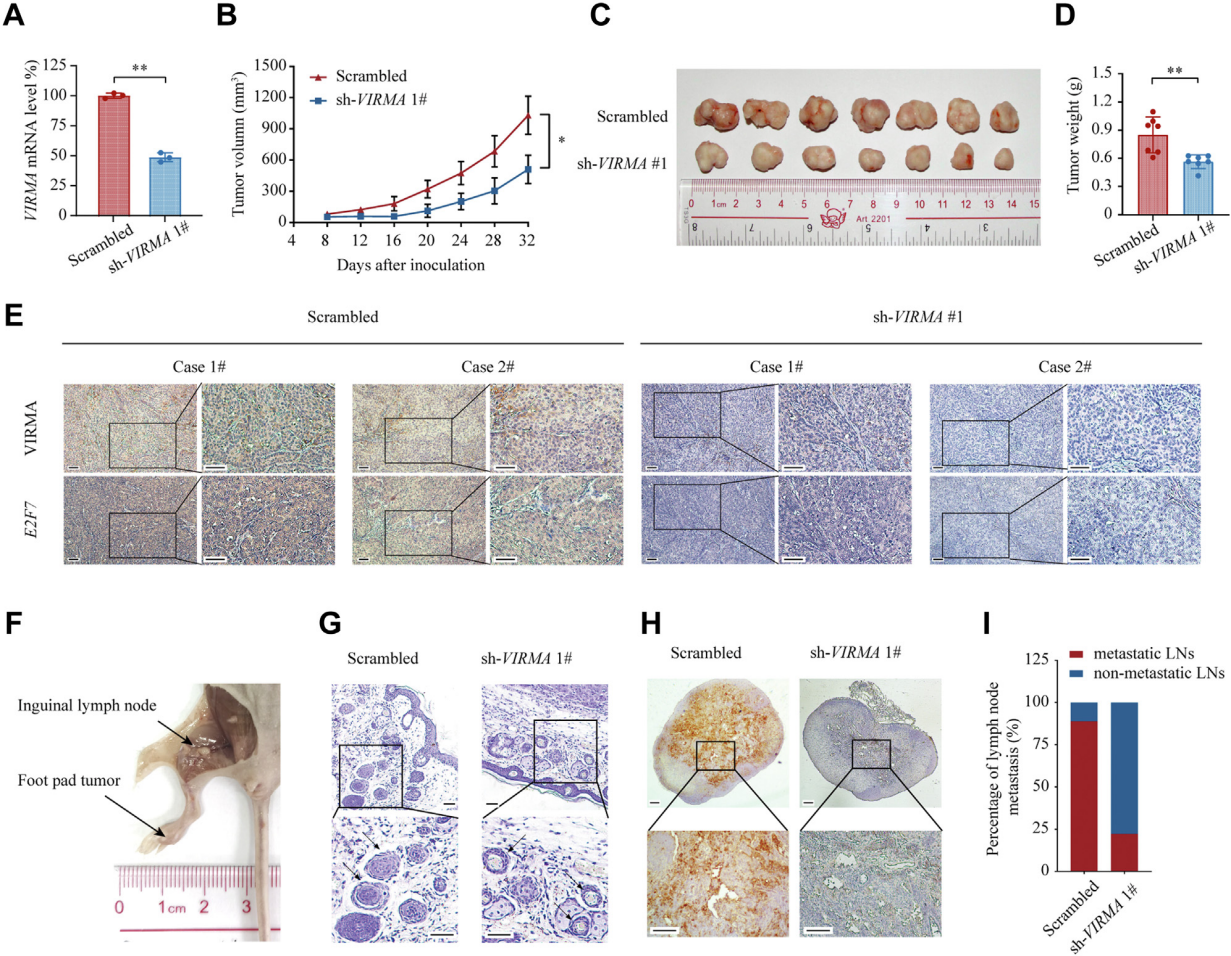
**Depletion of *VIRMA* impaired the proliferation and invasion of NPC *in vivo*.***A*, SUNE-1 cells were stably transduced with scrambled shRNA or sh-*VIRMA* 1# lentivirus, and quantitative RT-PCR was used for validation. *B*–*E*, subcutaneous tumorigenesis model. *B*, tumor volume of subcutaneous xenografts with or without *VIRMA* depletion was measured every 4 days during 5 weeks of growth. Subcutaneous xenograft tumors were retrieved from sacrificed mice on day 32 after axilla inoculation (*C*), and the tumor weight was measured (*D*). *E*, subcutaneous xenograft tumors were embedded in paraffin and cut into 5 μm sections, which were stained using IHC (VIRMA, *upper row*) and ISH (*E2F7*, *lower row*). Scale bar: 50 μm. *F*–*I*, SUNE-1 cells with or without *VIRMA* silencing were inoculated into the footpad of nude mice to establish the inguinal lymph node metastasis model. *F*, representative image of primary tumors (footpad) and metastatic inguinal lymph nodes in the inguinal lymph node metastasis model. *G*, representative images of primary tumor sections stained with hematoxylin and eosin showing tumor cells invasion into lymphatic vessels (*arrows*). Scale bar: 100 μm. *H*, representative images of pan-cytokeratin staining in inguinal lymph nodes. Scale bar: 100 μm. *I*, ratios of inguinal lymph nodes metastasis from primary tumors in the footpad. Data are presented as the mean ± SD. ∗*p* < 0.05, ∗∗*p* < 0.01. IHC, immunohistochemistry; ISH, *In situ* hybridization; NPC, nasopharyngeal carcinoma; VIRMA, vir-like m6A methyltransferase associated.

To further evaluate the tumor-promoting role of VIRMA in NPC *in vivo*, we used a subcutaneous tumorigenesis model and an inguinal lymph node metastasis model. First, *VIRMA*-depleted SUNE-1 cells and control cells were injected subcutaneously into nude mice separately (Fig. 3*A*). Notably, the depletion of *VIRMA* significantly decreased the growth rate of the xenografts (Fig. 3*B*). At 5 weeks post-inoculation, the mice were sacrificed. The volumes and weights of the tumors derived from the *VIRMA*-depleted group were substantially decreased compared with those from the control group (Fig. 3, *C* and *D*). We verified the downregulation of *VIRMA* in SUNE-1 cells bearing sh-*VIRMA* by immunohistochemistry (Fig. 3*E*). Next, we utilized the inguinal lymph node metastasis model to evaluate how VIRMA affects NPC aggressiveness *in vivo* (Fig. 3*F*). Depletion of *VIRMA* substantially impaired the invasive capacity of primary tumors into lymphatic vessels (Fig. 3*G*). To evaluate the infiltration of NPC cells in the inguinal lymph nodes, we utilized immunostaining on pan-cytokeratin (pan-CK), a specific epithelial cell marker to indicate metastatic tumor cells, for inguinal lymph node staining. Significantly, fewer pan-CK-positive tumor cells were observed in the *VIRMA*-depleted group than those in the control group, indicating that *VIRMA* silencing decreased the metastatic ratio in inguinal lymph nodes (Fig. 3, *H* and *I*). Taken together, these *in vivo* results suggested that VIRMA promotes an aggressive phenotype of NPC.

### *E2F7* functions as a crucial m6A target of VIRMA during NPC development

**Figure 4:**
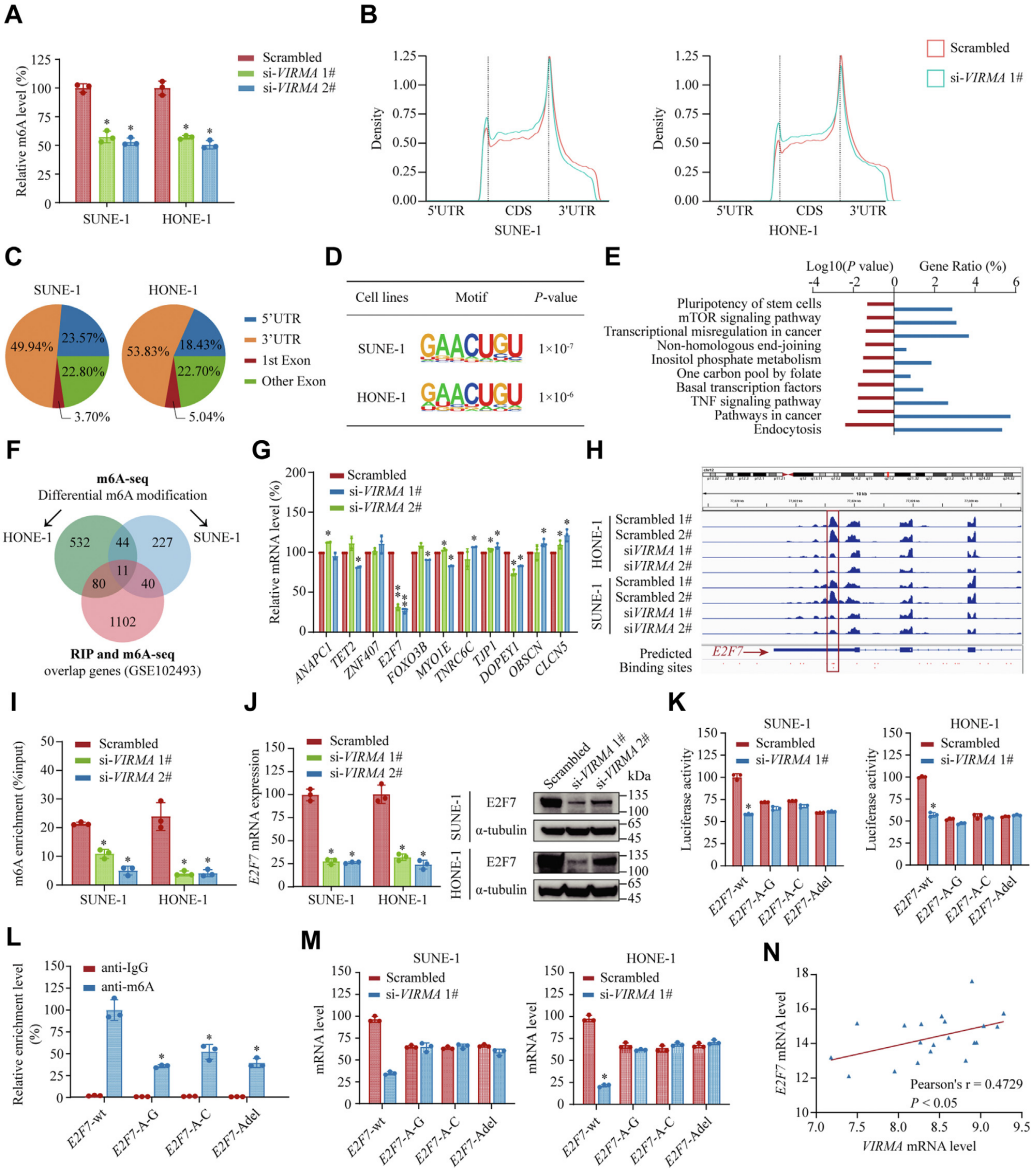
**VIRMA promotes m6A deposition on *E2F7* 3′ UTR and upregulates *E2F7* expression in NPC.***A*, the effects of *VIRMA* depletion on total polyadenylated RNA m6A modification levels were quantified using an ELISA-based m6A quantification assays (*A*). *B*, the profiles of m6A peak density along with RNA transcripts, with or without *VIRMA* silencing. *C*, top consensus motif identified by MeRIP-seq in SUNE-1 and HONE-1 cells. *D*, pie charts illustrating the proportion of m6A peaks distribution in the 5′ UTR, CDS, and 3′ UTR regions of the mapped transcripts. *E*, Kyoto Encyclopedia of Genes and Genomes (KEGG) pathway analysis of MeRIP-seq data in HONE-1 cells. *F*, Venn diagram of differential m6A modifications in SUNE-1 and HONE-1 cells upon *VIRMA* knockdown and the overlapping genes of VIRMA RIP-seq and m6A-seq in the GEO database (GSE102493). *G*, relative expression levels of 11 high-confidence VIRMA downstream candidates after *VIRMA* silencing analyzed by qRT-PCR. *H*, m6A peaks enriched in the 3′ UTR and around the stop codon of *E2F7*, with or without *VIRMA* knockdown in SUNE-1 and HONE-1 cells from the MeRIP-seq data visualized by Integrative Genomics Viewer (IGV) browser tracks. *I*, relative m6A enrichment in the E2F7 3′ UTR analyzed using MeRIP-qPCR in control and *VIRMA*-silencing cells. *J*, relative expression level of *E2F7* in *VIRMA*-deficient SUNE-1 and HONE-1 cells detected by qRT-PCR (*left panel*) and Western blotting (*right panel*). *K*, relative luciferase activity in SUNE-1 and HONE-1 cells transfected with *E2F7* 3′ UTR wild-type or mutant constructs upon *VIRMA* knockdown. Data are shown as the relative ratio of *Firefly* to *Renilla* luciferase activity. *L*, relative m6A enrichment of *E2F7* 3′ UTR fragments in HONE-1 cells transfected with *E2F7* 3′ UTR wild-type or mutant constructs analyzed by MeRIP-qPCR. *M*, relative expression levels of *E2F7* 3′ UTR fragments in SUNE-1 and HONE-1 cells transfected with *E2F7* 3′ UTR wild-type or mutant constructs, with or without *VIRMA* knockdown. *N*, Pearson correlation analysis of *VIRMA* and *E2F7* expression in 20 NPC tissues. Data are presented as the mean ± SD. ∗*p* < 0.05, ∗∗*p* < 0.01. Adel mut, mutant with adenine residues deletion; A mut G, mutant with A–G transition mutations; A mut T mut, mutant with A–T transversion mutations; m6A, N6-methyladenosine; NPC, nasopharyngeal carcinoma; VIRMA, vir-like m6A methyltransferase associated; WT, wild-type.

As a component of the methyltransferase complex, VIRMA was reported to mediate m6A methylation in 3′-untranslated regions (3′ UTR) and near-stop codons of downstream transcripts (21). Therefore, we tested whether VIRMA regulated global m6A methylation in NPC cells. As expected, enzyme-linked immunosorbent assay (ELISA)-based m6A quantification assays revealed that *VIRMA* silencing significantly decreased m6A modification (Fig. 4*A*). Then, we performed methylated RNA immunoprecipitation sequencing (MeRIP-seq) to identify potential downstream targets that might be essential in NPC. Consistent with previous reports (21), the m6A peaks were especially abundant in the 3′ UTR and around the stop codons. Knockdown of *VIRMA* primarily decreased m6A modification in the 3′ UTR and near stop codons (Fig. 4, *B* and *C*). The MeRIP-seq results also revealed that m6A modification was highly enriched in the consensus RRACH motif (R corresponding to A or G; H corresponding to A, C, or U), in both scrambled and *VIRMA* knockdown cells (Fig. 4*D*). Functional enrichment analysis showed that m6A-modified transcripts were highly enriched in cancer-related pathways, basal transcription factors, and transcriptional dysregulation in cancer (Fig. 4*E*), indicating that m6A modification has a pivotal role in promoting NPC proliferation and metastasis *via* controlling transcription regulatory networks in NPC. To identify high-confidence m6A-modified transcripts, we integrated data across (i) genes that showed significantly reduced m6A modification upon *VIRMA* silencing in both HONE-1 and SUNE-1 cells (|log2(fold change (FC))| > 2, *p* < 0.05) and (ii) genes that overlapped with VIRMA RNA immunoprecipitation sequencing (RIP-seq) targets and MeRIP-seq targets in HeLa cells (GSE102493). The multidimensional datasets revealed 11 high-confidence m6A-modified transcripts that may mediate VIRMA’s facilitating NPC cell tumorigenicity effects (Fig. 4*F*). We further screened the expression level of these potential targets and found that *E2F7*, an atypical transcription factor in the E2F family, was the most downregulated candidate upon *VIRMA* knockdown (Fig. 4*G*). MeRIP-seq showed that the m6A level on the 3′ UTR region of *E2F7* transcripts exhibited a substantial decrease in *VIRMA*-knockdown NPC cells compared with those in the control cells, which was further confirmed by MeRIP-qPCR (Fig. 4, *H* and *I*). In line with these findings, MeRIP-seq data from the GEO database (GSE102493) showed a dampened m6A peak in the 3′ UTR region of *E2F7* upon *VIRMA* silencing or mutation (Fig. S4*A*). Besides, we detected a marked decrease in *E2F7* expression at both mRNA and protein levels in SUNE-1 and HONE-1 cells after *VIRMA* knockdown (Fig. 4*J*). Consistently, we found that depletion of *VIRMA* substantially decreased *E2F7* expression *in vivo* (Fig. 3*E*).

To further investigate whether VIRMA regulates *E2F7* expression in an m6A-dependent manner, we constructed *E2F7* 3′ UTR mutant expression vectors, where adenine residues in the m6A consensus motif (RRACH) were replaced by guanine (A mut G) and cytosine (A mut C) or deleted (A del). *E2F7* 3′ UTR-reporter assays showed that knockdown of *VIRMA* largely reduced the luciferase activity of constructs harboring the wild-type (*E2F7*-wt) but not the mutated *E2F7* 3′ UTR (*E2F7*-A-G, *E2F7*-A-C, or *E2F7*-Adel) (Fig. 4*K*). Moreover, MeRIP-qPCR analysis showed that point mutation of adenine residues significantly decreased the m6A level of the *E2F7* 3′ UTR (Fig. 4*L*). More importantly, the knockdown of *VIRMA* significantly reduced the expression level of wild-type, but not the mutated, constructs (Fig. 4*M*) suggesting that VIRMA enhanced *E2F7* expression in an m6A-dependent manner.

E2F7 is a cellular context-dependent transcription factor that might act as a positive or negative regulator in different cancer types (22, 23). Its function in NPC remained uncharacterized. GEO data (GSE12452) analysis revealed that the expression of *E2F7* was significantly increased in NPC tissues compared with that in normal nasopharyngeal tissues (Fig. S4*B*). CCK-8 and Transwell assays revealed that silencing *E2F7* significantly decreased the proliferation ability and migration/invasion capacity of SUNE-1 and HONE-1 cells, similar to that of the *VIRMA*-knockdown treatment (Fig. S4, *C* and *D*). *VIRMA* expression was positively associated with *E2F7* expression in NPC tissues (Figs. 4*N* and S4*E*). Notably, we exogenously overexpressed wild-type E2F7 or E2F7-Adel mutant in NPC cells with *VIRMA* silenced, and found that overexpression of wild-type E2F7 could rescue the cell proliferation, migration, and invasion defects by *VIRMA* depletion, whereas the E2F7-Adel mutant could not (Fig. S4, *F*–*I*). These findings suggest that E2F7 functions as a facilitating factor of NPC cell tumorigenicity. Collectively, VIRMA promotes NPC proliferation and metastasis by targeting *E2F7*.

### IGF2BP2 mediates *E2F7* mRNA stability *via* an m6A-dependent mechanism

**Figure 5:**
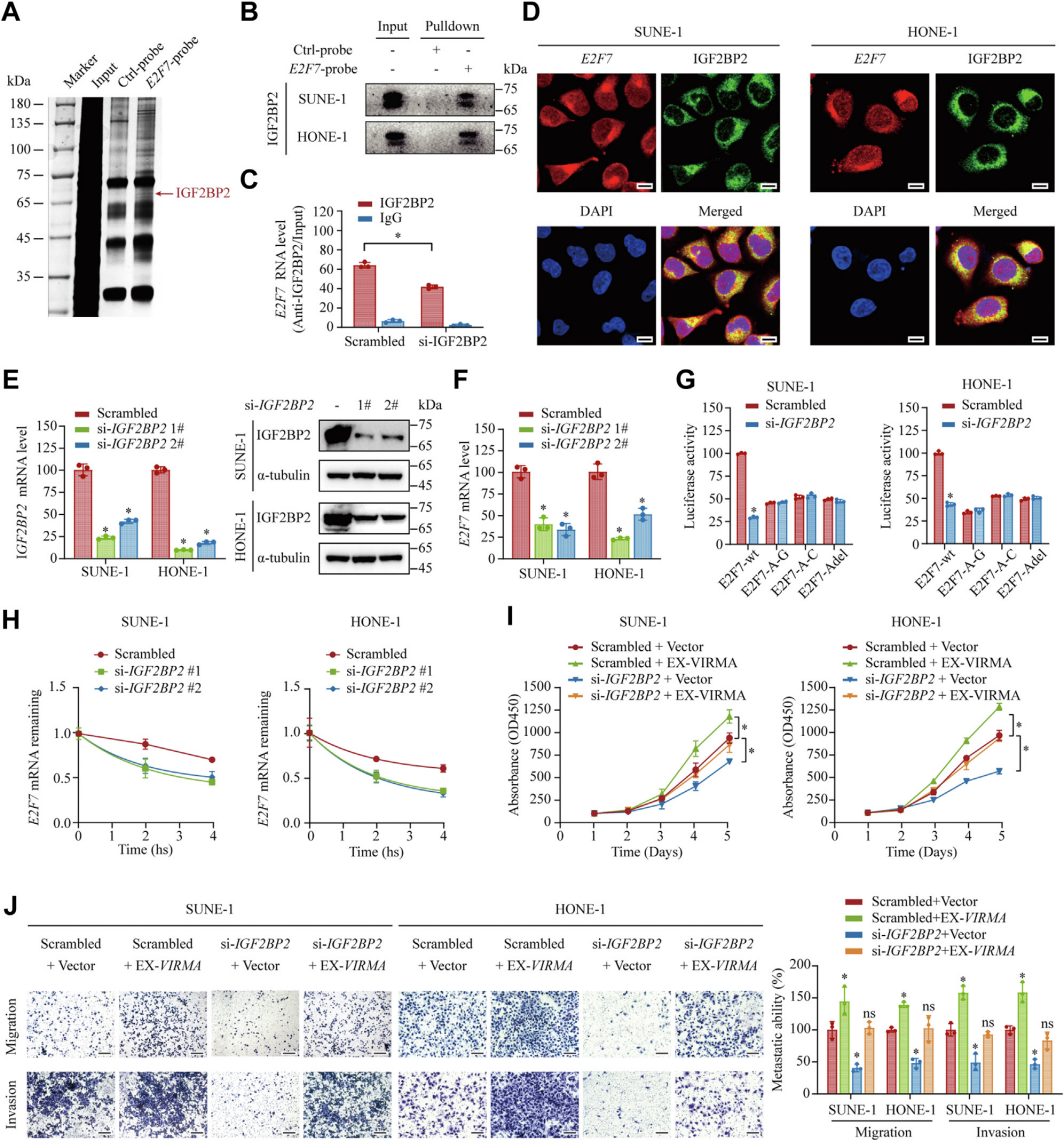
**IGF2BP2 maintains *E2F7* RNA stability and through an m6A-dependent mechanism.***A* and *B*, an RNA pulldown assay was conducted using a biotin-labeled *E2F7* probe and a control probe. The subsequently enriched proteins were subjected to silver staining (*A*) and Western blotting analysis (*B*). *C*, relative enrichment level of *E2F7* transcripts immunoprecipitated by anti-IGF2BP2 or anti-IgG antibodies. *D*, intracellular distribution of *E2F7* transcripts and IGF2BP2 visualized by FISH accompanied by IF. *Red*, *E2F7* transcripts; *Green*, IGF2BP2 proteins; *Blue*, DAPI. Scale bar: 10 μm. *E*, knockdown efficiency of *IGF2BP2* was validated using qRT-PCR (*left panel*) and Western blotting (*right panel*). *F*, relative mRNA expression level of *E2F7* in control or *IGF2BP2*-depleted SUNE-1 and HONE-1 cells. *G*, relative luciferase activity in SUNE-1 and HONE-1 cells transfected with wild-type or mutant *E2F7* 3′ UTR constructs upon *IGF2BP2* silencing. Data are shown as the relative ratio of *Firefly* to *Renilla* luciferase activity. *H*, *E2F7* RNA stability with or without *IGF2BP2* silencing in SUNE-1 and HONE-1 cells quantified by qRT-PCR. *I* and *J*, the effect of *IGF2BP2* knockdown on abrogating the VIRMA-mediated promotion of cell proliferation and invasion. CCK-8 assays (*I*) and Transwell assays (*J*) were performed, respectively, for cell proliferation and invasion. Scale bar: 200 μm. Data are presented as the mean ± SD. ∗*p* < 0.05, ∗∗*p* < 0.01. CCK-8, Cell Counting Kit-8; IGF2BP, insulin-like growth factor 2 mRNA-binding protein; VIRMA, vir-like m6A methyltransferase associated.

It has been reported that m6A methylation on mRNAs is recognized by m6A readers, which control the fate of m6A-modified transcripts (18). To explore the specific m6A readers of *E2F7* mRNA, we performed an RNA pulldown assay followed by Western blotting analysis. We found that *E2F7* mRNA–IGF2BP2 complexes were specifically precipitated using the *E2F7* mRNA probe rather than the control probe (Fig. 5, *A* and *B*). RIP assays further confirmed the interaction between *E2F7* mRNA and IGF2BP2 (Fig. 5*C*). To intuitively visualize the intracellular distribution of *E2F7* mRNA and IGF2BP2, we performed FISH combined with immunofluorescence (IF) assays and found that *E2F7* mRNA co-localized with IGF2BP2 in the cytoplasm (Fig. 5*D*). Collectively, IGF2BP2 directly interacted with *E2F7* mRNA, suggesting that IGF2PB2 might serve as an m6A reader of *E2F7*.

IGF2BP2 belongs to the IGF2BP family, which has been shown recently to interpret m6A methylation and promote mRNA stability (24). Then, we knocked down IGF2BP2 in SUNE-1 and HONE-1 cells to test whether the expression level of *E2F7* was regulated by IGF2BP2 (Fig. 5*E*). Compared with that in the control cells, silencing of *IGF2BP2* significantly inhibited the expression level of *E2F7* (Fig. 5*F*). However, the knockdown of *IGF2BP2* did not affect the expression level of *VIRMA* (Fig. S5*A*). Then, we investigated whether m6A modification in the 3′ UTR region of *E2F*7 was essential for IGF2BP2-mediated *E2F7* expression. Dual-luciferase reporter assays showed that silencing of *IGF2BP2* significantly reduced the luciferase activity in reporters harboring wild-type *E2F7* 3′ UTR fragments. Consistently, mutations in the consensus m6A motif completely abrogated this effect (Fig. 5*G*), suggesting that IGF2BP2 promoted *E2F7* expression in an m6A-dependent manner. We further investigated whether IGF2BP2 controlled the expression of *E2F7* by stabilizing its mRNA. As expected, mRNA stability analysis revealed that silencing *IGF2BP2* accelerated the degradation of *E2F7* transcripts (Fig. 5*H*).

In NPC tissues, *IGF2BP2* expression was positively associated with *E2F7* expression (Fig. S5*B*). In addition, the increased expression of *E2F7* in *VIRMA*-overexpressing cells was partially reversed by the knockdown of *IGF2BP2* (Fig. S5*C*). CCK-8 assays showed that *IGF2BP2* silencing impaired the growth-promoting effect of *VIRMA* overexpression in SUNE-1 and HONE-1 cells (Fig. 5*I*). Moreover, Transwell migration and invasion assays also revealed that the knockdown of *IGF2BP2* reversed the increased migration/invasion capacity of *VIRMA*-overexpressing NPC cells (Fig. 5*J*). Altogether, these results showed that VIRMA regulates *E2F7* expression *via* an m6A-dependent and IGF2BP2-mediated pathway.

### E2F7 cooperates with CBFB-recruited RUNX1 to transcriptionally activate *ITGA2*, *ITGA5*, and *NTRK1*

**Figure 6:**
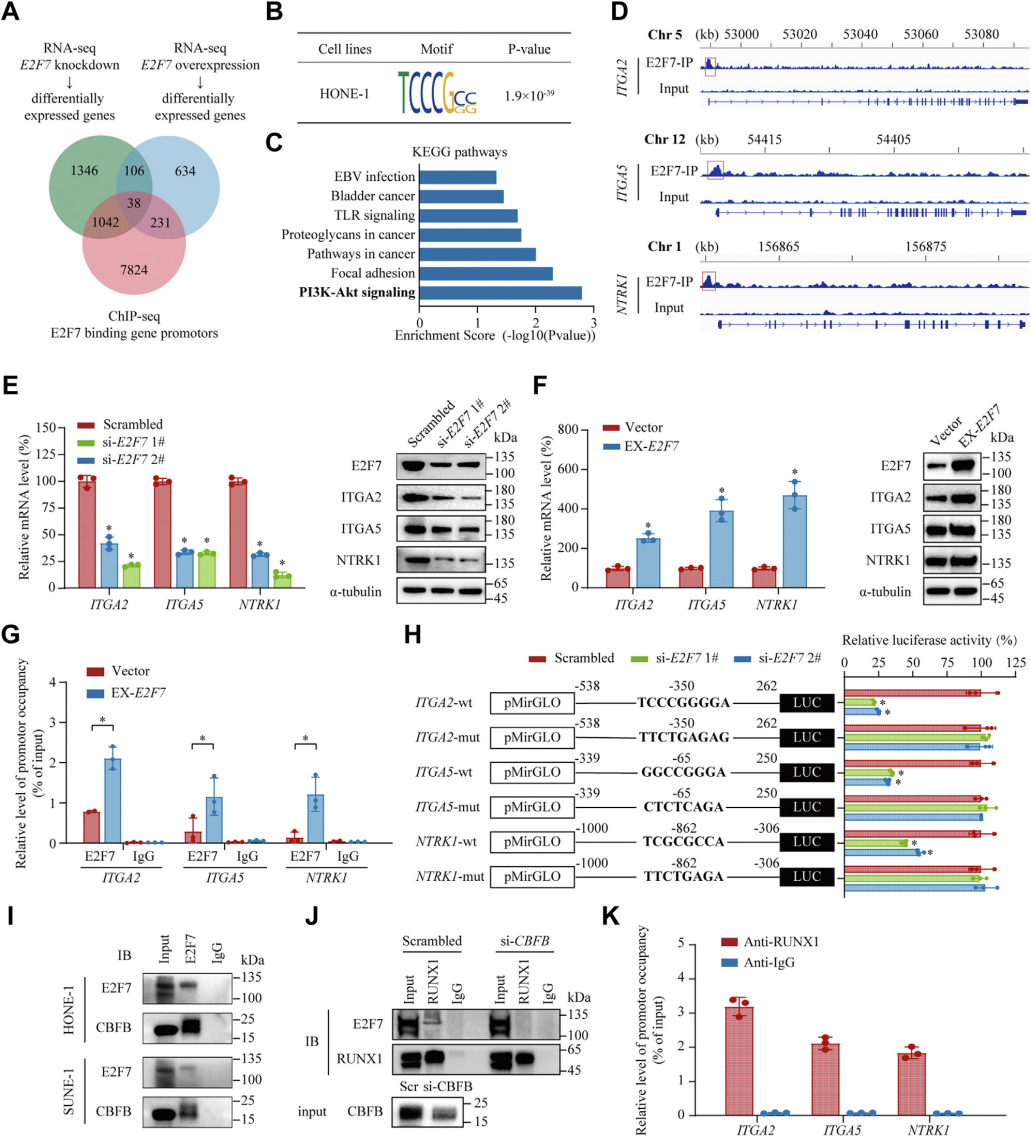
**E2F7 transactivates *ITGA2*, *ITGA5*, and *NTRK1* cooperatively with CBFB-recruited RUNX1.***A*, Venn diagram of differentially expressed genes after *E2F7* knockdown or *E2F7* overexpression identified by RNA-seq and E2F7 binding gene promoters identified by ChIP-seq. *B*, the top consensus motif was analyzed by the motif discovery algorithm DREME (https://meme-suite.org/meme/tools/dreme) based on the ChIP-seq data. *C*, Kyoto Encyclopedia of Genes and Genomes (KEGG) pathway analysis of differentially expressed genes identified by RNA-seq. *D*, ChIP-seq revealed E2F7 binding to promoter regions of *ITGA2*, *ITGA5*, and *NTRK1* in HONE-1. *E*, relative RNA (*left panel*) and protein (*right panel*) levels of *ITGA2*, *ITGA5*, and *NTRK1* upon *E2F7* knockdown. *F*, relataive RNA (*left panel*) and protein (*right panel*) levels of *ITGA2*, *ITGA5*, and *NTRK1* with or without *E2F7* overexpression. *G*, relative level of occupancy in *ITGA2*, *ITGA5*, and *NTRK1* promoter regions by E2F7, with or without *E2F7* overexpression. *H*, *left*, schematic representation of the *ITGA2*, *ITGA5*, and *NTRK1* promoter regions constructed on luciferase reporter expression vectors; *Right*, relative luciferase activity in HONE-1 cells transfected with wild-type or E2F7-binding-site mutant *ITGA2*, *ITGA5*, or *NTRK1* luciferase reporter expression vectors upon *E2F7* silencing. The numbers indicated the distance to transcription start sites (TSS) of the corresponding gene. *I*, Co-IP assays performed using anti-E2F7 antibodies or normal rabbit IgG to detect the interaction between endogenous E2F7 and CBFB in HONE-1 and SUNE-1 cells. *J*, immunoblotting of exogenous E2F7 and RUNX1 immunoprecipitated using anti-RUNX1 antibodies or normal rabbit IgG, with or without *CBFB* silencing, using immunoprecipitation assays. *K*, relative level of occupancy in the *ITGA2*, *ITGA5*, and *NTRK1* promoter regions by RUNX1. Data are presented as the mean ± SD. ∗*p* < 0.05. CBFB, Core-binding factor subunit beta; ITGA, integrin subunit alpha; mut, mutant; NTRK1, neurotrophic receptor tyrosine kinase; RUNX1, RUNX family transcription factor 1; WT, wild-type.

To gain an insight into the underlying mechanism by which E2F7 promotes NPC proliferation and metastasis, we performed RNA-seq, with or without *E2F7* knockdown or overexpression, and performed E2F7 ChIP-seq in HONE-1 cells. We identified 38 candidates that might be regulated directly by E2F7 (Fig. 6*A*). ChIP-seq analysis revealed that E2F7 binding sites were highly enriched in the E2F-related TCCCGCC motif (Fig. 6*B*), as reported previously (25). Besides, we found that E2F7-regulated genes were the most enriched in phosphatidylinositol-4,5-bisphosphate 3-Kinase (PI3K)-Akt signaling pathway through Kyoto Encyclopedia of Genes and Genomes analysis (Fig. 6*C*). We then screened the genes related to PI3K-Akt signaling pathway among the 38 candidates and identified three genes, *ITGA2* (encoding integrin α2), *ITGA5* (encoding integrin α5), and *NTRK1* (encoding the neurotrophic receptor tyrosine kinase 1). E2F7 is an atypical E2F transcription factor, which commonly antagonizes classical E2F activators, such as E2F1, and inhibits E2F-targeted gene expression (26, 27, 28). Unexpectedly, RNA-seq showed that E2F7 activated rather than repressed the expression levels of *ITGA2*, *ITGA5*, and *NTRK1*. Notably, E2F7 directly bound to the promoter region of these genes, indicating that E2F7 activates their transcription (Fig. 6*D*). To verify this finding, we examined the expression levels of *ITGA2*, *ITGA5*, and *NTRK1* upon *E2F7* knockdown or overexpression. Consistent with RNA-seq results, the silencing of *E2F7* markedly reduced the expression levels of *ITGA2*, *ITGA5*, and *NTRK1* (Fig. 6*E*). Accordingly, overexpression of *E2F7* significantly upregulated the expression levels of *ITGA2*, *ITGA5*, and *NTRK1* (Fig. 6*F*). Then, we tested whether E2F7 controlled *ITGA2*, *ITGA5*, and *NTRK1* expression through direct promoter regulation. By performing ChIP-qPCR assays, we observed significant enrichment of E2F7 in the promoter regions of *ITGA2*, *ITGA5*, and *NTRK1*. Besides, overexpression of *E2F7* specifically increased E2F7 binding to their promoters (Fig. 6*G*). Using JASPAR online software (29), we predicted E2F7-binding sites in the promoter regions of *ITGA2*, *ITGA5*, and *NTRK1*. Luciferase reporter assays revealed that the knockdown of *E2F7* significantly reduced the luciferase activity of the wild-type reporters. This reduction was diminished by mutation of the predicted E2F7-binding sites (Fig. 6*H*). These results suggested that E2F7 transcriptionally activated *ITGA2*, *ITGA5*, and *NTRK1* in NPC.

However, E2F7 lacks a transactivation domain (25). Thus, we hypothesized that E2F7 transactivated its target genes in cooperation with other transcriptional activators. To identify its binding partners, we immunoprecipitated E2F7 in HONE-1 cells and carried out mass spectrometry. Core-binding factor subunit beta (CBFB) was identified as a potential interacting partner of E2F7 (Table S2). Western blotting confirmed that CBFB was immunoprecipitated by anti-E2F7 antibodies (Fig. 6*I*). More importantly, the knockdown of *CBFB* significantly downregulated the expression of *ITGA2*, *ITGA5*, and *NTRK1* (Fig. S6*A*). However, the knockdown of *CBFB* did not alter the expression level or promoter binding ability of E2F7 (Fig. S6, *B* and *C*). CBFB was reported as a non-DNA binding regulatory subunit of the core-binding factor (CBF) complex that enhanced the DNA-binding capacity of RUNX1 (30). Therefore, we tested whether CBFB recruited RUNX1 to E2F7 and cooperatively activated transcription. As expected, Co-IP assays showed that E2F7 was immunoprecipitated using anti-RUNX1 antibodies. More importantly, silencing of *CBFB* markedly abolished the interaction between E2F7 and RUNX1 (Fig. 6*J*). Meanwhile, the knockdown of *RUNX1* reduced the expression levels of *ITGA2*, *ITGA5*, and *NTRK1* (Fig. S6*D*). To further validate that E2F7 transactivates *ITGA2*, *ITGA5*, and *NTRK1* in a RUNX1-dependent manner, we re-analyzed the proximal promoter regions of *ITGA2*, *ITGA5*, and *NTRK1* identified by ChIP-seq. Interestingly, the E2F7-binding sites were in close proximity to binding sites for RUNX-1. Consistently, ChIP-qPCR assays validated the enrichment of RUNX1 in these promoter regions (Fig. 6*K*). In addition, luciferase reporter assays revealed that mutations in the RUNX1-binding sites reversed the inhibition of the luciferase activity by *E2F7* silencing (Fig. S6*E*). These findings showed that the collaborative action of E2F7 with CBFB recruited RUNX1 transcriptionally activated the expression of *ITGA2*, *ITGA5*, and *NTRK1* by binding to their promoters.

### ITGA2, ITGA5, and NTRK1 were responsible for VIRMA-induced NPC progression

**Figure 7:**
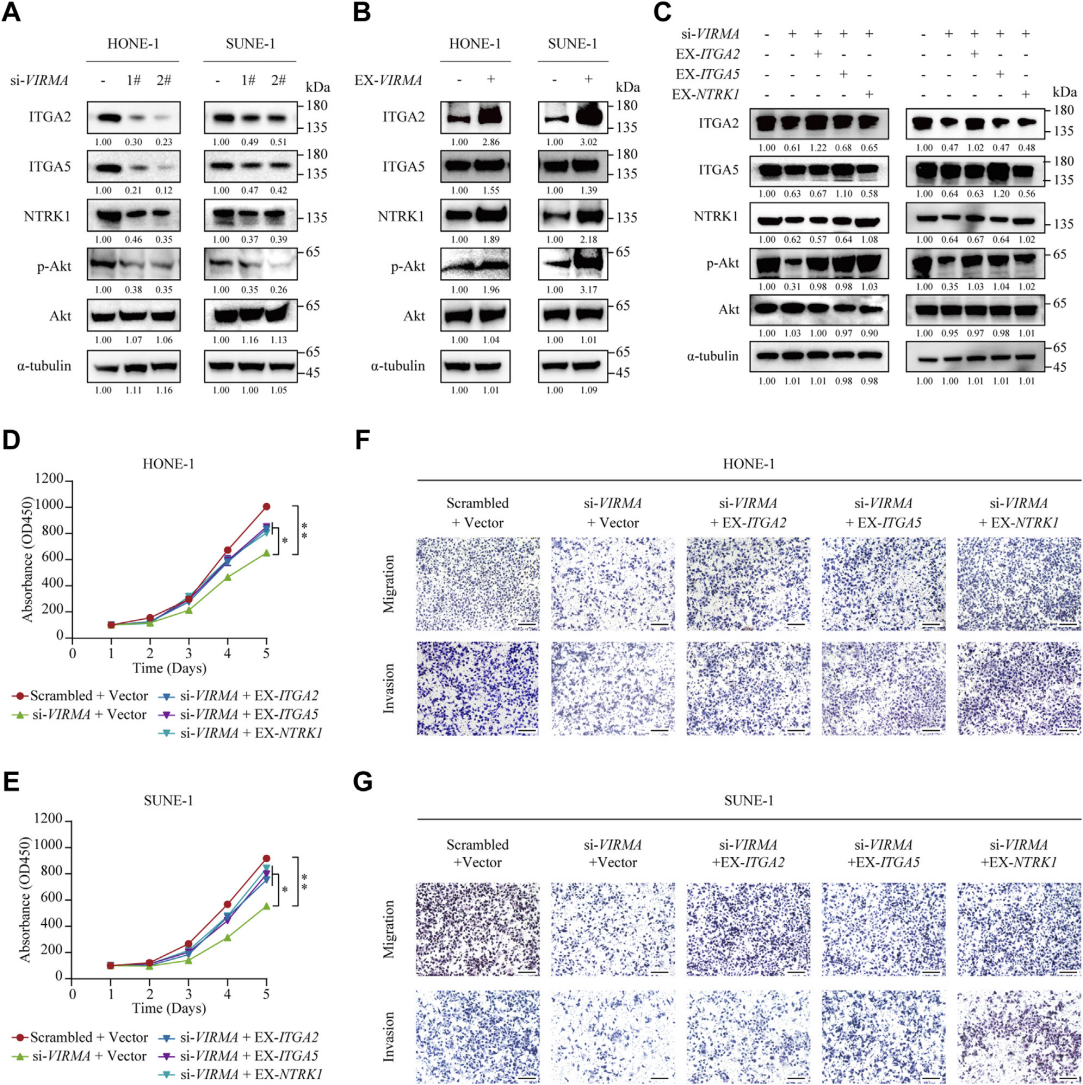
**VIRMA upregulates *ITGA2*, *ITGA5*, and *NTRK1* expression and activates the PI3K-Akt signaling pathway to promote NPC cell proliferation and metastasis.***A*, Western blotting analysis of ITGA2, ITGA5, NTRK1, phospho-Akt (p-Akt), and Akt in SUNE-1 and HONE-1 cells transfected with scrambled control or si-VIRMA. *B*, Western blotting analysis of ITGA2, ITGA5, NTRK1, p-Akt, and Akt in SUNE-1 and HONE-1 cells transfected with empty vectors or VIRMA overexpressing vectors. *C*, Western blotting analysis of ITGA2, ITGA5, NTRK1, p-Akt, and Akt in SUNE-1 and HONE-1 cells co-transfected with scrambled control or si-*VIRMA*, together with the empty vector, or *ITGA2*, *ITGA5*, or *NTRK1* overexpression vectors. *D* and *E*, CCK-8 assays determining the proliferation ability of SUNE-1 (*D*) and HONE-1 (*E*) cells co-transfected with scrambled control or si-*VIRMA*, together with empty vector, *ITGA2*, *ITGA5*, or *NTRK1* overexpression vectors. *F* and *G*, Transwell assays to evaluate the migration and invasion capacity of SUNE-1 (*F*) and HONE-1 (*G*) cells co-transfected with scrambled control or scrambled control or si-*VIRMA*, together with empty vector, *ITGA2*, *ITGA5*, or *NTRK1* overexpression vectors. Scale bar: 200 μm. Data are presented as the mean ± SD. ∗*p* < 0.05. CCK-8, Cell Counting Kit-8; ITGA, integrin subunit alpha; NTRK1, neurotrophic receptor tyrosine kinase; RUNX1, RUNX family transcription factor 1; VIRMA, vir-like m6A methyltransferase associated.

**Figure 8:**
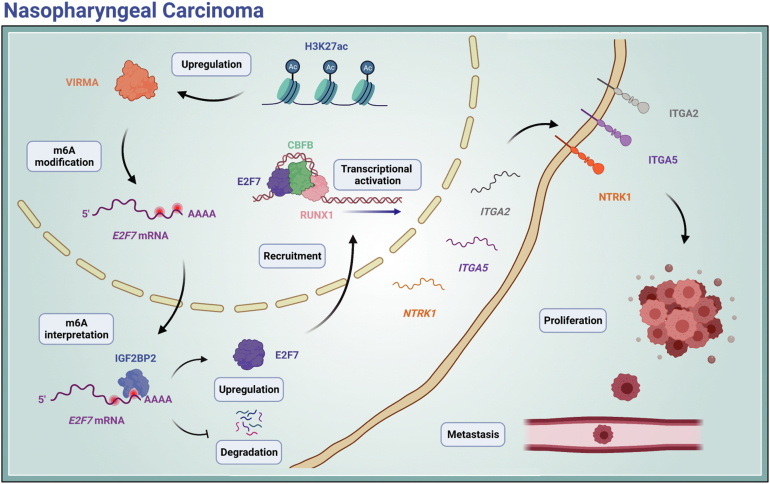
**The diagram of VIMRA promotes tumorigenesis and metastasis in an m6A-dependent manner.***VIRMA* was transcriptionally activated by KAT3A-mediated H3K27ac. Upregulation of VIRMA substantially increased m6A deposition in *E2F7* mRNA, which promoted *E2F7* expression in an m6A-IGF2BP2-dependent manner. Ultimately, E2F7 interacted with CBFB, then recruited RUNX1 to transcriptionally activate *ITGA2*, *ITGA5*, and *NTRK1*, and endowed proliferative and metastatic properties for NPC cells. ITGA, integrin subunit alpha; m6A, N6-methyladenosine; NPC, nasopharyngeal carcinoma; NTRK1, neurotrophic receptor tyrosine kinase; VIRMA, vir-like m6A methyltransferase associated.

ITGA2, ITGA5, and NTRK1 have been reported to promote tumorigenesis by activating the PI3K-Akt signaling pathway (31, 32, 33). We first tested whether VIRMA modulated the PI3K-Akt pathway by regulating the expression levels of ITGA2, ITGA5, and NTRK1 in NPC. Silencing *VIRMA* significantly downregulated the protein levels of ITGA2, ITGA5, and NTRK1 as well as the level of phosphorylated Akt (Fig. 7*A*). Accordingly, overexpression of *VIRMA* increased ITGA2, ITGA5, and NTRK1 levels and simultaneously promoted downstream Akt activation (Fig. 7*B*). Rescue experiments showed that overexpression of ITGA2, ITGA5, or NTRK1 could partially abrogate the inhibited Akt activation by VIRMA knockdown (Fig. 7*C*), suggesting that VIRMA promotes ITGA2, ITGA5, and NTRK1 expression, which consequently activates the PI3K-Akt pathway. To investigate whether VIRMA exerts its tumor-promoting function through upregulating ITGA2, ITGA5, and NTRK1 expression, we performed *in vitro* functional experiments by overexpressing ITGA2, ITGA5, or NTRK1 in *VIRMA*-silenced NPC cells. CCK-8 assays showed that overexpression of ITGA2, ITGA5, or NTRK1 mitigated the repressed cell proliferation induced by VIRMA silencing (Fig. 7, *D* and *E*). Transwell migration and invasion assays showed that overexpression of ITGA2, ITGA5, or NTRK1 abolished the inhibitory cell metastatic ability induced by VIRMA knockdown (Fig. 7, *F* and *G*). These findings demonstrated that the VIRMA-E2F7 axis promotes NPC proliferation and metastasis by upregulating ITGA2, ITGA5, and NTRK1 expression, which subsequently activates the PI3K/Akt pathway (Fig. 8).

## Discussion

In the present study, we identified VIRMA as a vital modulator in NPC. *VIRMA* was transcriptionally activated by KAT3A-mediated H3K27ac. Upregulation of VIRMA substantially increased m6A deposition in *E2F7* mRNA, which promoted E2F7 expression in an m6A-IGF2BP2-dependent manner. Ultimately, E2F7 interacted with CBFB, then recruited RUNX1 to transcriptionally activate ITGA2, ITGA5, and NTRK1, and endowed proliferative and metastatic properties for NPC cells.

In most situations, human tumor cells harbor abnormalities of epigenetic modification, suggesting that these abnormal changes can promote tumorigenesis (34). While m6A and histone modification are both essential for tumor growth and development, their interplay remains elusive yet (35). Therefore, a deep investigation of m6A and histone modification interaction provides novel insights into the epigenic regulation in NPC. In this study, we showed that the transcription of *VIRMA* is aberrantly activated because of enriched H3K27ac, an active histone mark, within its promoter region in NPC. It is still noteworthy that reciprocal regulation between m6A and histone modification is widespread in tumor cells. A recent study revealed that METTL14 identified H3K36me3 mark and recruited the m6A methyltransferase complex to co-transcriptionally deposit the m6A modification (36). Reciprocally, m6A modifications guide histone modification through m6A-binding proteins. YTHDC1 mediates H3K9me2 demethylation through recruiting lysine demethylase 3B (KDM3B) to m6A-marked chromatin regions (37). Our findings reveal an upstream mechanism to account for the dysregulation of m6A regulator, providing evidence that histone modifications regulate the expression of m6A regulators and further control global m6A levels in cancer cells. The regulatory network between m6A and histone modifications in NPC needs to be further determined.

RNA m6A modification is regulated precisely by a cluster of m6A regulators. While emerging evidence suggests that m6A regulators serve as critical drivers or checkpoints in various types of cancer (38, 39), studies on the role of m6A regulators and m6A-dependent mechanisms in NPC are limited. Here, we revealed that VIRMA acted as a tumor-promoting gene to enhance NPC growth and metastasis in an m6A-dependent way. As one of the m6A methyltransferase components, VIRMA was initially identified to preferentially mediate m6A deposit in 3′ UTR and near stop codon of mRNA (21). Subsequently, numerous research proved that VIRMA was abnormally highly expressed in multiple cancer types and contributed to tumor progression. Moreover, in line with our results, high VIRMA expression was indicative of poor prognosis (40, 41, 42). These findings indicated that VIRMA can be exploited as a biomarker of cancer progression and as a target for anti-cancer therapies. Indeed, targeting the m6A pathways possessed strong potential against tumor progression and was recognized as one of the most promising anticancer targets (43). Small molecule inhibitors of *FTO*, FB23, and FB23-2, strongly inhibited human acute myeloid leukemia cell growth, showing a powerful antitumor effect (44). STM2457, the first *METTL3* inhibitor, was validated to reduce the m6A deposit on several oncogenes (such as *HOXA10* and *MYC*), induce cell differentiation, and effectively inhibit the growth of leukemia cells (45). However, no effective use of drugs targeting m6A regulators has been reported in NPC. Our findings offer the conceptual basis and experimental foundation for the development of novel anti-NPC medicine.

E2F7 is a cellular context-dependent transcription factor that might act as a positive or negative regulator in different cancer types (22, 23). Recent studies have reported that E2F7 is upregulated significantly and promotes tumorigenesis in various cancer types (22, 46, 47, 48, 49). In the present study, we found that *E2F7* mRNA is a *bona fide* target of VIRMA and promotes cell proliferation and metastasis in NPC. Since E2F7 lacks a transactivation domain, it commonly acts as a direct transcriptional repressor of classical E2F transcription factor, such as E2F1 (25, 28). Through antagonizing the effect of classical E2F proteins, E2F7 participates in a wide range of biological processes, including cell proliferation, apoptosis, and DNA damage repair (25). Surprisingly, integrated ChIP-seq and RNA-seq revealed that several known oncogenes were directly activated instead of repressed by E2F7 in NPC. A previous study reported that E2F7 associates with hypoxia-inducible factor 1 (HIF1) and activates the transcription of VEGFA (encoding vascular endothelial growth factor A) to promote angiogenesis (50). Thus, we hypothesized that E2F7 transactivated oncogenes with the help of partner proteins in a non-canonical manner. Interestingly, we did not identify classical E2F proteins as being immunoprecipitated using anti-E2F7 antibodies. Instead, we found that E2F7 formed a transcription complex with CBFB and RUNX1. CBFB is a non-DNA binding subunit of a heterodimeric transcription factor belonging to the PEBP2/CBF family (30, 51). While previous studies have suggested that CBFB enhances the DNA binding capacities of RUNX1 (30), we did not observe the increased DNA binding ability of E2F7 upon CBFB silencing. However, the knockdown of CBFB abolished the interaction between E2F7 and RUNX1, suggesting that CBFB serves as a scaffold or adaptor for the E2F7-CBFB-RUNX1 complex. As a result, E2F7 cooperates with CBFB-recruited RUNX1 to transactivate *ITGA2*, *ITGA5*, and *NTRK1* in NPC.

## Conclusion

These data supported a model whereby VIRMA maintains transcription factor *E2F7* mRNA stability *via* an m6A-dependent mechanism. Consequently, increased E2F7 interacts with CBFB-recruited RUNX1 to form a transcription complex, which cooperatively transactivates the expression of target genes *ITGA2*, *ITGA5*, and *NTRK* and eventually promotes NPC progression and metastasis. This study reveals the importance of VIRMA-mediated m6A modifications in NPC progression and metastasis and provides *VIRMA* as a prognostic biomarker and promising therapeutic target for NPC management.

## Experimental procedures

### Patients and clinical specimens

This study was approved by the Institutional Ethical Review Board of Sun Yat-Sen University Cancer Center (GZR2021-129). Written informed consent was obtained from all patients prior to the study. All human paraffin-embedded NPC tissues, which had not been treated with chemotherapy or radiotherapy before biopsy, were obtained from patients with detailed clinical characteristics and long-term follow-up data from January 2004 to December 2013. All cancers were verified pathologically.

### Cells and culture conditions

All cell lines had been authenticated and were generously provided by Dr M. Zeng (Sun Yat-sen University Cancer Center). Human NPC cells were cultured in Roswell Park Memorial Institute-1640 medium (Invitrogen) or Dulbecco’s modified Eagle’s medium (Invitrogen) supplemented with 10% fetal bovine serum (FBS; Invitrogen). The human immortalized nasopharyngeal epithelial cell lines, including NP69, N2Tert, and N2Bmil, were maintained in keratinocyte serum-free medium (Invitrogen) containing bovine pituitary extract (BD Biosciences). Cells were grown in a 5% CO_2_ incubator at 37 °C.

### RNA extraction, reverse transcription, and quantitative real-time PCR

Total RNA from cells and tissues was extracted using the TRIzol Reagent (Invitrogen). The cDNAs were synthesized using HiScriptIII RT SuperMix (Vazyme). The quantitative real-time PCR (qPCR) assays were conducted using the cDNA as the template with a CFX96 Touch Real-Time PCR Detection System (Bio-Rad, Hercules) with Platinum SYBR Green qPCR SuperMix-UDG reagents (Invitrogen). The threshold cycle number (CQ) was analyzed in triplicate for each sample. *GAPDH* (encoding glyceraldehyde-3-phosphate dehydrogenase) was used as the internal control for CQ value normalization. The primer sequences used for qPCR are shown in Table S3.

### Western blotting

Cells were lysed in Radioimmunoprecipitation assay buffer (Millipore) containing protease and phosphatase inhibitors (Thermo Fisher Scientific) to extract the total protein. Protein concentrations were determined using a bicinchoninic acid Protein Assay Kit (Thermo Fisher Scientific). Western blotting analysis was performed as described in our previous study (16). The antibodies used in this study recognized VIRMA (ab271136, 1:1000, Abcam), IG2BP2 (ab188200, 1:2000, Abcam), E2F7 (ab245655, 1:1000, Abcam), ITGA2 (ab181548, 1:1000, Abcam), ITGA5 (ab150361, 1:1000, Abcam), NTRK1 (ab76291, 1:1000, CST), protein kinase B (Akt; #4685,1:1000, CST), phospho-Akt (#4060, Ser473, 1:1000, CST), CBFB (ab133600, 1:1000, Abcam), RUNX1 (ab240639, 1:1000, Abcam), and α-tubulin (ab7291, 1:5000, Abcam).

### Plasmid construction, cell transfection, and lentiviral infection

For RNA knockdown, the synthesized duplex RNA interference (RNAi) oligos targeting human *KAT3A* (encoding CREB binding protein), *VIRMA*, and *IGF2BP2* mRNA sequences were purchased from RiboBio (Table S4). A scrambled duplex RNA oligo was adopted as an RNA-negative control. A specific short hairpin RNA (shRNA) against VIRMA or a scrambled shRNA was designed and cloned into the pLKO.1 vector (Addgene). Specific primers are listed in Table S5. The 3′ UTR of *E2F7* and m6A methylated site mutations (wild type, A-G, A-C, Adel) were synthesized by Genscript and cloned into the pcDNA3.1 (−) or pmir-GLO vectors to generate overexpression or dual-luciferase vectors. An empty vector was used as a control.

Cells were transiently transfected with siRNAs using Lipofectamine RNAiMAX (Invitrogen) or with plasmids using Lipofectamine 3000 (Invitrogen) according to the manufacturer’s instructions. For *VIRMA* stable knockdown, lentiviruses expressing shRNA targeting *VIRMA* or scrambled shRNA control were co-transfected with lentivirus packaging plasmids pMD2G and psPAX2 (Addgene) into the human embryonic kidney (HEK) 293T cells. The supernatant containing lentivirus was collected after 48 h of incubation and filtered. SUNE-1 and HONE-1 cells were infected with the virus solution accordingly to the manufacturer’s instructions. Target cells were selected and maintained using 1 μg/ml puromycin (Thermo Fisher Scientific).

### Chromatin immunoprecipitation

The chromatin immunoprecipitation assay was performed using a Pierce Magnetic ChIP Kit (Thermo Fisher Scientific) according to the manufacturer’s instructions. The antibodies used for immunoprecipitation were anti-H3K27ac (CST), anti-E2F7 (Abcam), and anti-RUNX1 (Abcam). Purified DNA was detected with specific primers for the promoter region using quantitative PCR. The primer sequences used for quantitative PCR are shown in Table S6.

### Cell proliferation and colony formation assays

For the cell proliferation assay, 1 × 10^3^ cells were seeded per well in a 96-well plate, and cell viability was detected every 24 h for 5 days using a Cell Counting Kit-8 (CCK-8, Dojindo) according to the manufacturer's instructions. For the colony formation assay, 400 cells were seeded per well in 6-well plates and cultured for 10 to 14 days until colonies were detected. Cell colonies were then rinsed, fixed, stained, and counted.

### Transwell migration and invasion assay

For the Transwell invasion and migration assays, 5 × 10^4^ and 1 × 10^5^ cells in serum-free media were seeded into the upper chambers, respectively, for migration (without Matrigel) and invasion (with Matrigel) assays. 10% FBS-containing medium was added to the lower chamber. After 12 or 24 h, the cells were fixed, stained, and counted under an inverted microscope.

### Global m6A quantification and m6A dot blotting

Total RNA was extracted using TRIzol and subjected to mRNA separation using a GenElute mRNA Miniprep Kit (Sigma). Global m6A levels in polyadenylated RNAs were measured using an EpiQuik m6A RNA Methylation Quantification Kit (Epigentek) following the manufacturer’s instructions. Briefly, an equal amount of polyadenylated RNAs (200 ng) were coated on the assay wells. Antibody capture solution and antibody detection solution were added sequentially to the assay wells. The m6A levels were measured and calculated based on a standard curve. The m6A dot blotting was conducted as previously described (52). Polyadenylated RNA samples were loaded into a nitrocellulose membrane (Boster-Bio) fixed with 96-wells Bio-Dot Module (Bio-Rad) and cross-linked using a UV crosslinker. After blocking and incubating with anti-m6A antibodies (1:500; Synaptic Systems), the membrane was incubated with horseradish peroxidase (HRP)-conjugated secondary antibodies and then detected using the ECL detection system (Bio-Rad).

### Methylated RNA immunoprecipitation sequencing (MeRIP-seq) and MeRIP-qPCR

Total RNA was isolated from scrambled or *VIRMA*-silenced SUNE-1 and HONE-1 cells and analyzed using a Bioanalyzer 2100 and RNA 6000 Nano LabChip Kit (Agilent) with a RNA integrity number (RIN) number >7.0. After RNA purification and fragmentation, RNA samples were incubated with anti-m6A primary antibody (Synaptic Systems) for immunoprecipitation using a Magna MeRIP m6A kit (Millipore) according to the manufacturer’s instructions. Enriched m6A-modified RNA was then eluted and subjected to qRT-PCR or next-generation sequencing using the Illumina Novaseq 6000 platform (Illumina) at LC-BIO Biotech Ltd. Primer sequences are shown in Table S3.

### Dual-luciferase reporter assay

NPC cells were co-transfected with a *Firefly* luciferase reporter construct and the *Renilla* luciferase construct (Promega). Both luciferase activities were determined 48 h post-transfection using a Dual-luciferase Reporter Assay System (Promega) according to the manufacturer’s instructions. The Firefly luciferase activity was normalized to the *Renilla* luciferase activity.

### RNA pulldown assay

The biotin-labeled RNA pulldown probes were designed and synthesized by Sangon Biotech. RNA pulldown assays were conducted using Pierce Magnetic RNA-Protein Pull-Down Kit. Biotin-labeled probes were incubated with cell lysates and then pulled down using streptavidin-coated magnetic beads (Invitrogen) according to the manufacturer’s instructions. Bound proteins were eluted using an elution buffer and subjected to Western blotting or mass spectrometry (Fitgene Biotech) analysis. Silver staining was performed using Fast Silver Stain Kit (Beyotime) The probe sequences are shown in Table S7.

### RNA immunoprecipitation

The RNA immunoprecipitation (RIP) assay was performed using a Magna RIP Kit (Millipore) under the manufacturer’s instructions. For the RIP assay, cell lysates were incubated with Protein A/G magnetic beads coated with 10 μg of anti-IGF2BP2 antibody or normal IgG at 4 °C for 2 h. The immunoprecipitated RNAs were purified for qRT-PCR, and the primers are listed in Table S3.

### FISH and IF

FISH and IF co-staining were performed to detect the co-localization of *E2F7* mRNA and IGF2BP2 protein. Alexa Fluor 555-labeled *E2F7* FISH probes were purchased from RiboBio. After fixation and permeabilization, cells were incubated with the *E2F7* probes and then anti-IGF2BP2 antibodies (1:100, Abcam). Nuclei were stained with 4′,6-diamidino-2-phenylindole (DAPI). Images were captured using an Olympus confocal microscope (Olympus FV1000).

### Measurement of RNA stability

RNA decay assays were conducted to assess RNA stability. Briefly, NPC cells were seeded in 12-well plates and cultured until confluent. Then, actinomycin D (HY-17559, MedChemExpress) was added into each well to a final concentration of 10 μg/ml. Total RNAs were extracted at different time points (0, 1, 2, 3, and 4 h) and subjected to quantitative PCR to evaluate the abundance of E2F7 mRNA and 18S rRNA (normalized to time 0). The 18S rRNA was used as a control RNA whose expression remained unaffected by actinomycin treatment.

### Immunoprecipitation

The immunoprecipitation (IP) assay was performed using a Pierce Co-Immunoprecipitation Kit (Thermo Fisher Scientific). Cells were lysed with IP buffer supplemented with Protease/Phosphatase Inhibitor Cocktail. Cell lysates were pre-cleared and incubated with specific antibodies or normal IgG antibodies at 4 °C overnight with gentle rotation. Immunocomplexes were recovered and washed three times in IP buffer and then harvested for Western blotting or mass spectrometry (Fitgene Biotech) analysis.

### *In vivo* tumor xenograft models

All the experimental animal procedures were approved by the Institutional Animal Care and Use Ethics Committee of Sun Yat-sen University Cancer Center (L102012021008J). The 4-week-old BALB/c nude mice (female) were purchased from the Charles River Laboratories and were randomly divided into four groups before experiments. For the tumor growth model, 1 × 10^6^ SUNE-1 cells stably expressing scrambled or sh-*VIRMA* were injected subcutaneously into the axilla of mice, and the tumor size was measured every 4 days. After 32 days, the mice were sacrificed, and the tumors were retrieved. For the tumor inguinal lymph node metastasis model, 1 × 10^6^ scrambled or sh-*VIRMA* SUNE-1 cells were injected into the footpads of mice. After 6 weeks, the mice were euthanized. The footpad tumors and inguinal lymph nodes were excised. Tumors and lymph nodes were subjected to subsequent *in situ* hybridization and immunohistochemistry analysis.

### *In situ* hybridization

Specific *in situ* hybridization (ISH) probes for human *E2F7* were synthesized from Sangon Biotech. The ISH assay was conducted using an ISH Detection Kit (Boster-Bio) as described in our previous study (53). Briefly, samples were deparaffinized, rehydrated, and digested with pepsin at 37 °C for 10 min. Sections were hybridized with the *E2F7*-specific probes at 37 °C overnight and then incubated with corresponding antibodies. Then, sections were stained with streptavidin-HRP and visualized with 3,3′-diaminobenzidine. The probe sequence is listed in Table S8.

### Immunohistochemistry

For the IHC staining assay, sections were deparaffinized, rehydrated, inactivated for endogenous peroxidase activity, subjected to antigen retrieval, and blocked as described in our previous study (53). Then, sections were incubated with anti-VIRMA antibodies (1:200, Abcam) or anti-pan-cytokeratin antibodies (Thermo Fisher Scientific) at 4 °C overnight, followed by incubation with the corresponding secondary antibodies. For IHC staining analysis, an IRS system was used, as previously described (54). Briefly, the staining intensity was classified as non-existent (0), weak (1), moderate (2), or strong (3), and the proportion of cell staining was scored as no staining (0), less than 25% stained (1), 25 to 50% stained (2), 51 to 75% stained (3) or more than 75% stained (4). The IRS was calculated by multiplying these two variables. For statistical analysis, cases were grouped as either VIRMA low (IRS 0–6) or VIRMA high (IRS 7–12) expressions.

### Statistical analyses

All experiments were performed independently in triplicate. Statistical analyses were conducted using GraphPad Prism version 8.0 (GraphPad Inc), SPSS version 23.0 (IBM Corp), and R (version 3.6) software. Data are presented as the mean ± standard deviation (SD). Student’s *t* test or the *chi*-square test were used to compare differences between the two groups. One-way ANOVA followed by the Bonferroni test was used for multiple comparisons. Survival curves were assessed with the Kaplan–Meier method and compared using the Log-rank test. Univariate and multivariate Cox regression analyses were performed to calculate hazard ratios and 95% confidence intervals. Correlations were performed by Pearson correlation analysis. A *p* value of <0.05 was considered statistically significant.

## Data availability

The corresponding authors provided the data used and analyzed in this article upon request. The source data that support the findings of this study are available from the corresponding author upon request. MeRIP-seq, RNA-seq and ChIP-seq data are accessible at the GEO repository (GSE226363).

## Supporting information

This article contains supporting information.

## Conflict of interest

The authors declare that they have no conflicts of interest with the contents of this article.
