# Supplementary material for: VIRMA promotes nasopharyngeal carcinoma, tumorigenesis, and metastasis by upregulation of E2F7 in an m6A-dependent manner
Source: J Biol Chem. 2023 Apr 5;299(5):104677. doi: 10.1016/j.jbc.2023.104677 (PMC10235437; doi:10.1016/j.jbc.2023.104677)
Supplement: Supporting Tables S1–S8 [file mmc1.pdf]

Supplementary Table S1. Clinical characteristics of nasopharyngeal carcinoma patients according to the high and low expression of VIRMA

**Characteristics**	**No. of patients**	**Expression of VIRMA**		***P* value**
		**Low, *n* (%)**	**High, *n* (%)**	
**Age**				
≤45	82	46 (54.8)	36 (45.6)	0.274
>45	81	38 (45.2)	43 (54.4)	
**Sex**				
Male	121	62 (73.8)	59 (74.7)	1.000
Female	42	22 (26.2)	20 (25.3)	
**VCA-IgA**				
≤ 1:160	74	39 (46.4)	35 (44.3)	0.875
> 1:160	89	45 (53.6)	44 (55.7)	
**EA-IgA**				
≤ 1:20	87	45 (53.6)	42 (53.2)	1.000
> 1:20	76	39 (46.4)	37 (46.8)	
**T Stage**				
T1-T2	68	37 (44.0)	31 (39.2)	0.634
T3-T4	95	47 (56.0)	48 (60.8)	
**N Stage**				
N0-N1	101	54 (64.3)	45 (59.5)	0.628
N2-N3	62	30 (35.7)	32 (40.5)	
**TNM Stage**				
I-II	43	20 (23.8)	23 (29.1)	0.480
III-IV	120	64 (76.2)	56 (70.9)	
**Locoregional failure**				
Yes	22	7 (8.3)	15 (19.0)	0.065
No	141	77 (91.7)	64 (81.0)	
**Distant metastasis**				
Yes	22	6 (7.1)	17 (21.5)	**0.012**
No	139	78 (92.9)	62 (78.5)	
**Death**				
Yes	34	8 (9.5)	26 (32.9)	**0.001**
No	129	76 (90.5)	53 (67.1)	

VCA-IgA: viral capsid antigen immunoglobulin A; EA-IgA: early antigen immunoglobulin A. All patients were restaged according to the 8th edition of the AJCC Cancer Staging Manual. Bold values indicate $P < 0.05$, P value is determined by χ^2 and Fisher's exact tests.

Supplementary Table S2. The top 20 proteins found by mass spectrometry analysis in the E2F7 group

**Protein**	**Score**	**Matched peptides**
Transcription factor E2F7, E2F7	3632	113
Poly [ADP-ribose] polymerase 1, PARP1	1112	45
X-ray repair cross-complementing protein 6, XRCC6	955	32
X-ray repair cross-complementing protein 5, XRCC5	875	44
Plectin, PLEC	616	27
Transcription factor A, mitochondrial, TFAM	352	20
Ezrin, EZR	315	13
DNA ligase 3, LIG3	287	14
Serine/arginine-rich splicing factor 1, SRSF1	250	11
Clathrin heavy chain 1, CLTC	245	11
Putative elongation factor 1-alpha-like 3, EEF1A1P5	221	7
4F2 cell-surface antigen heavy chain, SLC3A2	212	6
Protein S100-A7, S100A7	207	6
Polyadenylate-binding protein 1, PABPC1	187	8
Moesin, MSN	185	10
Complement C4-A, C4A	175	3
Radixin, RDX	169	9
**Core-binding factor subunit beta, CBFB**	167	2
Sodium/potassium-transporting ATPase subunit alpha, ATP1A1	167	7
Heat shock protein beta-1, HSPB1	166	5

Supplementary Table S3. qRT-PCR primers used in this study

Gene	Sequence (5' to 3')
VIRMA-F	TGACCTTGCCTCACCAACTGCA
VIRMA-R	AGCAACCTGGTGGTTTGGCTAG
KAT3A-F	AGTAACGGCACAGCCTCTCAGT
KAT3A-R	CCTGTCGATACAGTGCTTCTAGG
E2F7-F	TCTGAACCCGACTGTCCCTCTT
E2F7-R	TTTGGCAGCCACATCCAGAGTG
ANAPC1-F	CAACATGGCAGCTCTAAGTCG
ANAPC1-R	AACTTTTGCCCACATAGGTCA
TET2-F	GCTTACCGAGACGCTGAGGAAA
TET2-R	AGAGAAGGAGGCACCACAGGTT
ZNF407-F	CCGCAATGAAAGACCACTACAGG
ZNF407-R	TGTGCTGTCTGCGATGCTTGGT
FOXO3B-F	AAGTGGCTGCTCTGCGCCTC
FOXO3B-R	AAGCCGGTGCCTCTGCCATCTT
MYO1E-F	CCACTACATTCGCTGCATCAAGC
MYO1E-R	AGGCATAGCCAGCTCTTCTCAC
TNRC6C-F	CACCGTTGCTTGGTCCAGTTTC
TNRC6C-R	TGCTGTGCCATTATCCACCAGG
TJP1-F	GTCCAGAATCTCGGAAAAGTGCC
TJP1-R	CTTTCAGCGCACCATACCAACC
DOPEY1-F	GTCCAAACCAGGGAAGAGTAGC
DOPEY1-R	CATCTCCCAACTGGTCCCACAA
OBSCN-F	CAGCTCCATTGTCAGGGTGCAT
OBSCN-R	GGACGTTGTTTCCATAGCACCAC
CLCN5-F	GTATCTGTAGCCTTTGGAGCACC
CLCN5-R	GGCAGCAAAGAATGAACGCCAC
E2F7-m^{6}A-F	TGCTGTAAATGAACTGTGCTA

(for MeRIP-qPCR)	
E2F7-m^{6}A-R (for MeRIP-qPCR)	TTCTCAAGCCCACAGTCAGG
IGF2BP2-F	GTTGGTGCCATCATCGGAAAGG
IGF2BP2-R	TGGATGGTGACAGGCTTCTCTG
ITGA2-F	TCGTGCACAGTTTTGAAGATG
ITGA2-R	TGGAACACTTCCTGTTGTTACC
ITGA5-F	GTCGGGGGCTTCAACTTAGAC
ITGA5-R	CCTGGCTGGCTGGTATTAGC
NTRK1-F	CACTAACAGCACATCTGGAGACC
NTRK1-R	TGAGCACAAGGAGCAGCGTAGA
GAPDH-F	TGATGACATCAAGAAGGTGG
GAPDH-R	TTGTCATACCAGGAAATGAGC

Supplementary Table S4. siRNA sequence used in this study

Name	Sequence (5' to 3')
si-VIRMA-1#-F	CCAUCAUCUUUAGACCUAATT
si-VIRMA-1#-R	UUAGGUCUAAAGAUGAUGGTT
si-VIRMA-2#-F	GCUGAUCACGUAUCAUCUUTT
si-VIRMA-2#-R	AAGAUGAUACGUGAUCAGCTT
si-KAT3A-1#-F	GCACAGCCGUUUACCAUGATT
si-KAT3A-1#-R	UCAUGGUAAACGGCUGUGCTT
si-KAT3A-2#-F	GCUCUAUAAUCGCAAGACATT
si-KAT3A-2#-R	UGUCUUGCGAUUAUAGAGCTT
si-E2F7-1#-F	GCAAAUGGCCUACCUCCAATT
si-E2F7-1#-R	UUGGAGGUAGGCCAUUUGCTT
si-E2F7-2#-F	GCAUCUGUCUUACCAGAAUTT
si-E2F7-2#-R	AUUCUGGUAAGACAGAUGCTT
si-IGF2BP2-1#-F	CAUGCCGCAUGAUUCUUGATT
si-IGF2BP2-1#-R	UCAAGAAUCAUGCGGCAUGTT
si-IGF2BP2-2#-F	AACAGGGACCAAGAUAACATT
si-IGF2BP2-2#-R	UGUUAUCUUGGUCCCUGUUTT
si-CBFB-F	CAGGAACCAAUCUGUCUCUTT
si-CBFB-R	AGAGACAGAUUGGUUCCUGTT

si-RUNX1-F	GGCAGAAACUAGAUGAUCATT
si-RUNX1-R	UGAUCAUCUAGUUUCUGCCTT

Supplementary Table S5. Primers used for shRNA plasmid construction

Name	Sequence (5' to 3')
sh-VIRMA-1#-F	CCGGTCGCCAACCTATCGCCTTAAACTCGAGTTTAAGGCGATAGGTTGGCGATTTTTG
sh-VIRMA-1#-R	AATTCAAAAATCGCCAACCTATCGCCTTAAACTCGAGTTTAAGGCGATAGGTTGGCGA
sh-VIRMA-2#-F	CCGGTACGCTCCTTTACACGATAAACTCGAGTTTATCGTGTAAAGGAGCGTATTTTTG
sh-VIRMA-2#-R	AATTCAAAAATACGCTCCTTTACACGATAAACTCGAGTTTATCGTGTAAAGGAGCGTA
sh-VIRMA-3#-F	CCGGAGGAGTGATCAGTGGATTATTCTCGAGAATAATCCACTGATCACTCCTTTTTTG
sh-VIRMA-3#-R	AATTCAAAAAAGGAGTGATCAGTGGATTATTCTCGAGAATAATCCACTGATCACTCCT

Supplementary Table S6. Primers sequence used for ChIP-PCR assay

Name	Sequence (5' to 3')
VIRMA-ChIP-F1	ATTACTTCTGTCAATGGCACT
VIRMA-ChIP-R1	GTCTAAATGGCACAAACCCTT
VIRMA-ChIP-F2	GGTTCATTCTTCGTACCTGCT
VIRMA-ChIP-R2	GCAGGAAACATAAGTGCCAT
VIRMA-ChIP-F3	TTAGACTACCAAACCCGCTGA
VIRMA-ChIP-R3	ATTGTGTAACGTGAAGTACCAT
VIRMA-ChIP-F4	TGTTTCCTGCTCTTTTGCAAC
VIRMA-ChIP-R4	TGGAGCAACGAGATAAACTCA
ITGA2-ChIP-F	GGAGCCGGGCGCTGCCAA
ITGA2-ChIP-R	ACTCCGCCCCCGCAGCCTG
ITGA5-ChIP-F	TCCTCACTCACCCGTCTGTTCCC
ITGA5-ChIP-R	AGGCCCCAGCAGTACTCTCG
NTRK1-ChIP-F	TTCCCTTTCTCTGCCCCGTCT
NTRK1-ChIP-R	AGGACTACCAAAGGATCAATCGC

Supplementary Table S7. Probe sequence of E2F7 RNA pulldown assays

**Name**	**Sequence (5' to 3')**
E2F7-pulldown-1	GUAUAGUUUGGCGACUUAAUGCACC-Biotin
E2F7-pulldown-2	AGCUGCAUUCUCUUAGUAGGACCAC-Biotin
E2F7-pulldown-3	AUAGAUGCGUCUCCUUUCCACACCA-Biotin
E2F7-pulldown-4	UAAAGAGUAGCCACCUGAUCCUUGU-Biotin

Supplementary Table S8. Probe sequence of E2F7 *in situ* hybridization (ISH)

Name	Sequence (5' to 3')
E2F7-ISH-1	UUCUAAAGAGUAGCCACCUGAUCC-DIG*(Biotin)
E2F7-ISH-2	AAUCUGUAAAUGCAUCGUCCUUGU-DIG(Biotin)
E2F7-ISH-3	ACUGUGUUAAAAGAACCAUGGCGA-DIG(Biotin)
E2F7-ISH-4	GCCAUACUGAUUCUUAGCCACCCG-DIG(Biotin)

*DIG: digoxin
