# Supplementary material for: VIRMA promotes nasopharyngeal carcinoma, tumorigenesis, and metastasis by upregulation of E2F7 in an m6A-dependent manner
Source: J Biol Chem. 2023 Apr 5;299(5):104677. doi: 10.1016/j.jbc.2023.104677 (PMC10235437; doi:10.1016/j.jbc.2023.104677)
Supplement: Supporting Figures S1–S6 [file mmc2.pdf]

Supplementary Figure 1

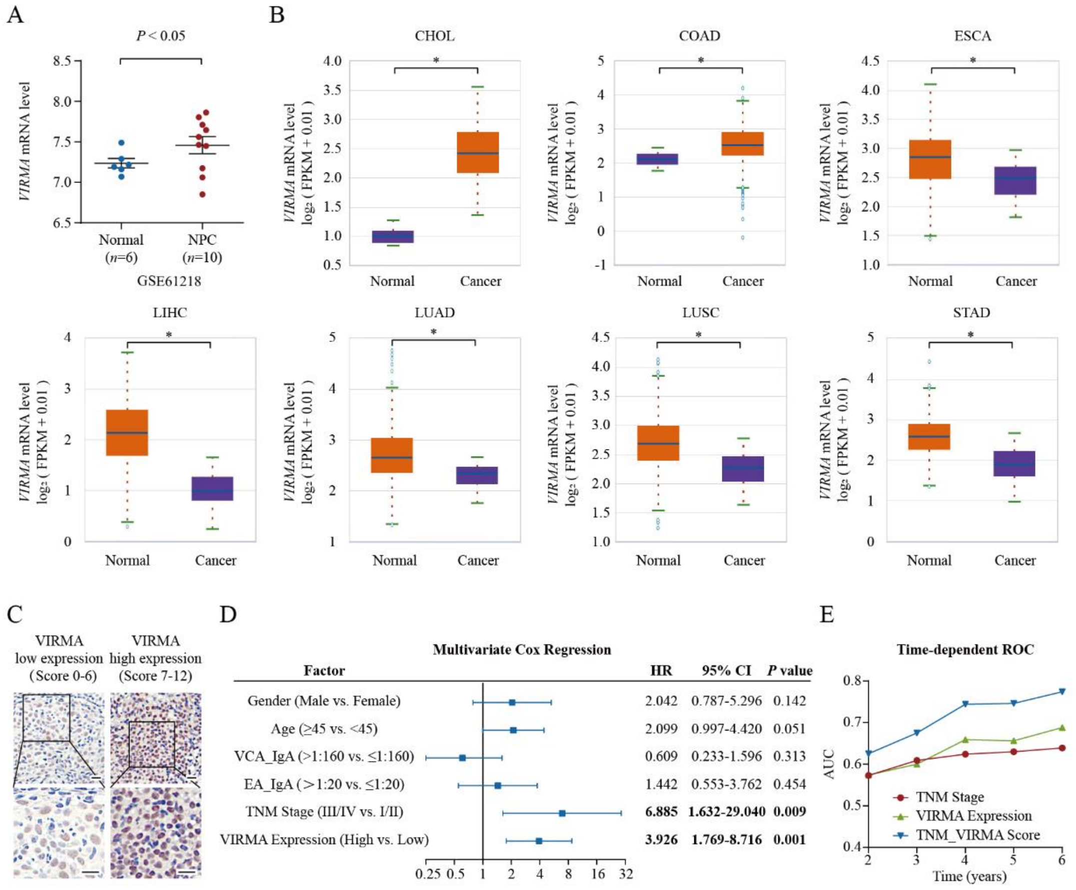

Supplementary Figure S1. *VIRMA* is upregulated in various cancer types and predicts an unfavorable prognosis for patients with NPC.

A. Expression levels of *VIRMA* in NPC tissues and normal nasopharyngeal tissues were compared based on the GEO database GSE61218. **B**. *VIRMA* expression levels in various cancer types based on RNA-seq data from TCGA databases. Abbreviations: CHOL, cholangiocarcinoma; COAD, colon adenocarcinoma; ESCA, esophageal carcinoma; HNSC, head and neck squamous cell carcinoma; LIHC, liver hepatocellular carcinoma; LUAD, lung carcinoma; LUSC, lung squamous carcinoma; STAD, stomach adenocarcinoma. **C**. Representative images of VIRMA low (score 0-6) and high (score 8-12) expression stained with IHC assays. Scale bar: 50 μm. **D.** Multivariate Cox regression was used to analyze the prognostic value of VIRMA expression. **E.** Time-dependent ROC analysis to determine the predictive ability of VIRMA expression of overall survival in NPC patients.

Supplementary Figure 2

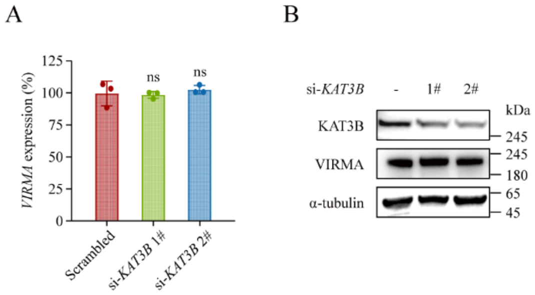

Supplementary Figure S2. Knockdown of KAT3B has no impacts on *VIRMA* expression.

A-B. The Relative expression of VIRMA was examined by qPCR (**A**) and western blotting (**B**) in SUNE-1 cells transfected with si-NC (scrambled control) or si-KAT3B (1#, 2#). Data presented as mean ± SD, ns = not significant.

Supplementary Figure 3

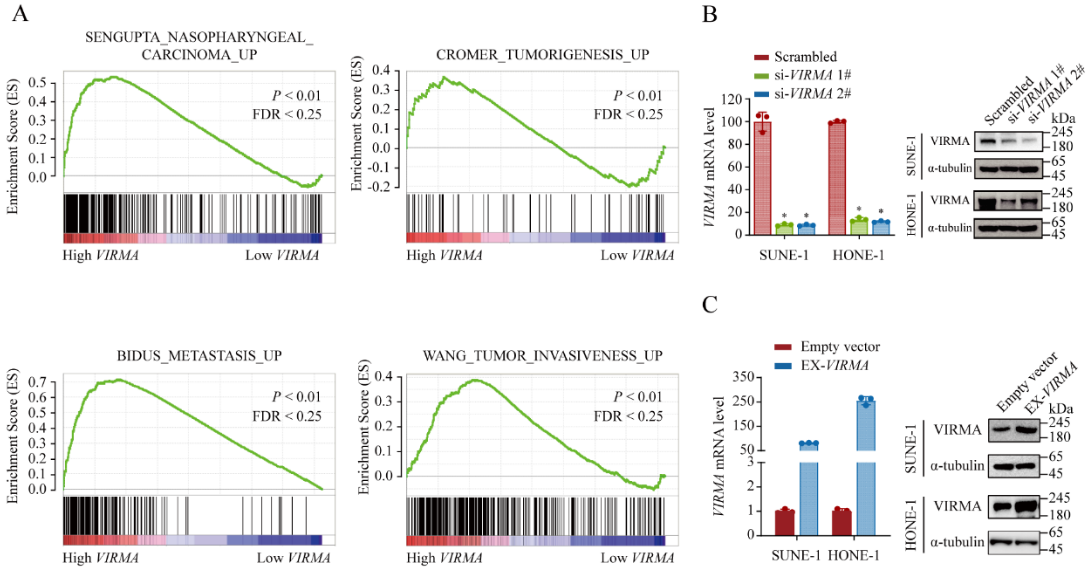

Supplementary Figure S3. Overexpression of *VIRMA* promotes NPC progression and metastasis.

A. GSEA analysis of RNA-seq data from GSE12452 revealed that *VIRMA* was closely associated with NPC tumorigenesis and metastasis. **B–C.** Knockdown (**B**) and overexpression (**C**) efficiency of *VIRMA* was validated by qPCR (left panel) and western blotting (right panel). Data are presented as the mean ± SD. *$P < 0.05$.

Supplementary Figure 4

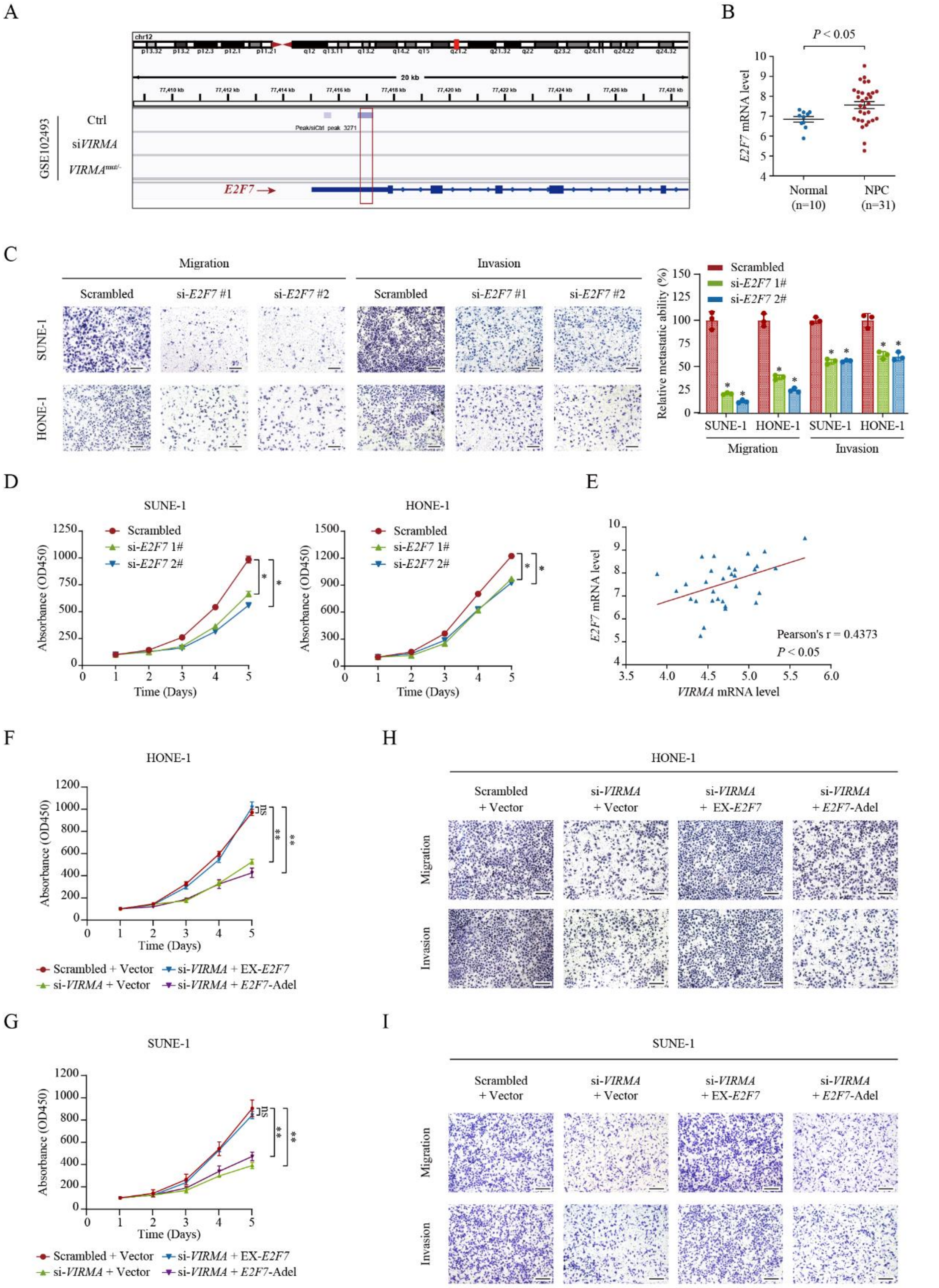

Supplementary Figure S4. VIRMA-mediated upregulation of *E2F7* promotes NPC proliferation and metastasis in NPC.

A. m6A peaks in the 3'UTR region of *E2F7* in control, *VIRMA* silencing or *VIRMA*-mutant Hela cells from the GEO database (GSE102493). **B.** Expression levels of *E2F7*

in NPC tissues and normal nasopharyngeal tissues were compared using the GEO database (GSE12452). **C.** Migration and invasion capacities of *E2F7*-silencing SUNE-1 and HONE-1 cells were analyzed by Transwell assays. Scale bar: 200 μm. **D.** CCK-8 assays were performed to analyze the proliferation ability of SUNE-1 and HONE-1 cells with *E2F7* silencing. **E.** Pearson correlation analysis of *VIRMA* and *E2F7* expression from GEO database (GSE12452). (**F–I**) HONE-1 and SUNE-1 cells co-transfected with scrambled control or si-*VIRMA*, together with the empty vector, *E2F7* overexpression vector, or *E2F7*-Adel expressing vector. Then, CCK-8 assays were used to determine the proliferation ability of HONE-1 (**F**) and SUNE-1 (**G**) cells. Transwell migration and invasion assays were used to assess the migration and invasion abilities of HONE-1 (**H**) and SUNE-1 (**I**) cells. Scale bar: 200 μm. Data are presented as the mean ± SD. *$P < 0.05$.

Supplementary Figure 5

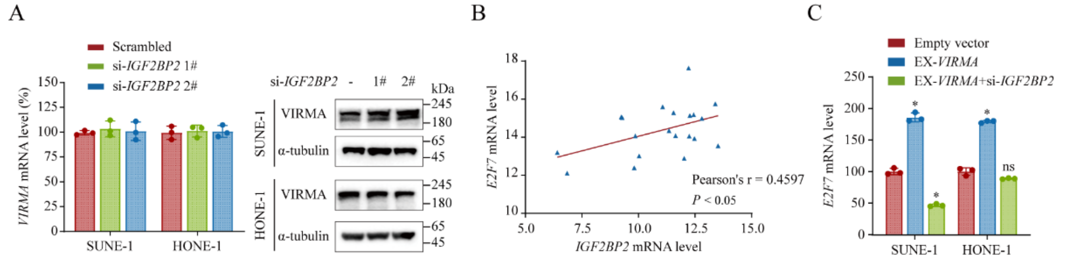

Supplementary Figure S5. VIRMA-mediated m6A modification of E2F7 mRNA maintains its IGF2BP2-dependent stability.

A. Quantitative RT-PCR (left) and western blotting (right) analysis of *VIRMA* expressions in SUNE-1 and HONE-1 cells with or without *IGF2BP2* silencing. **B.** Pearson correlation analysis of *IGF2BP2* and *E2F7* expression in 20 NPC tissues. **C.** Relative *E2F7* expression level in SUNE-1 and HONE-1 cells co-transfected with *VIRMA* overexpression vectors and si-*IGF2BP2* or the corresponding control. Data are presented as the mean ± SD. *$P < 0.05$, **$P < 0.01$.

Supplementary Figure 6

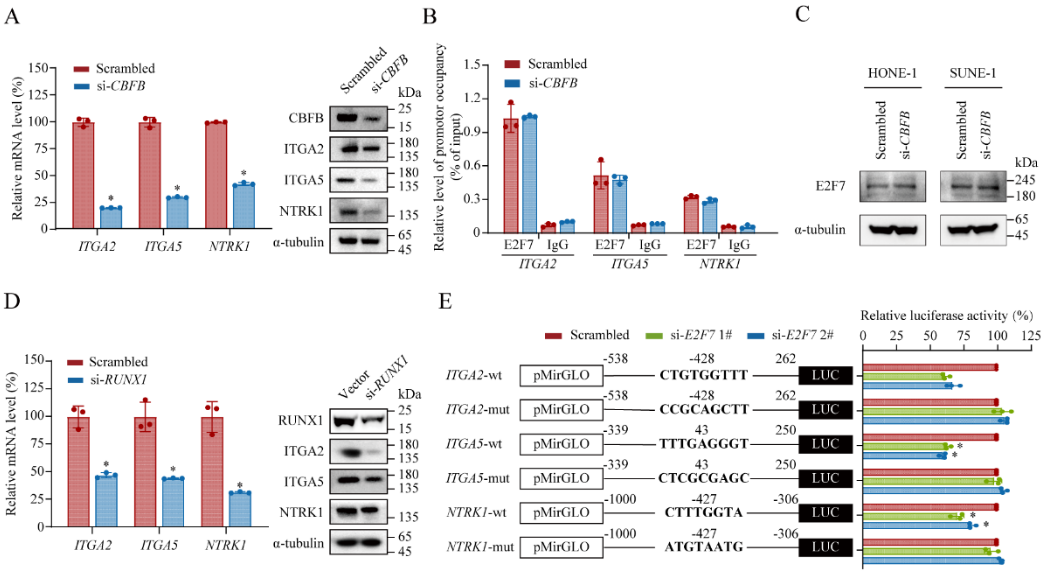

Supplementary Figure S6. E2F7 transactivates *ITGA2*, *ITGA5*, and *NTRK1* cooperatively with RUNX1.

A. Relative RNA (left panel) and protein (right panel) expression level of *ITGA2*, *ITGA5*, and *NTRK1* upon *CBFB* knockdown. **B.** Relative level of occupancy in *ITGA2*, *ITGA5*, and *NTRK1* promoter regions by E2F7 with or without *CBFB* knockdown. **C.** Relative E2F7 expression upon *CBFB* knockdown. **D.** Relative RNA (left panel) and protein (right panel) expression level of *ITGA2*, *ITGA5*, and *NTRK1* upon *RUNX1* knockdown. **E.** Left, schematic presentation of *ITGA2*, *ITGA5*, and *NTRK1* promoter regions constructed on luciferase reporter expression vectors; Right, relative luciferase activity in HONE-1 cells transfected with wild type or RUNX1-binding-site mutant *ITGA2*, *ITGA5* or *NTRK1* luciferase reporter expression vectors upon *E2F7* silencing. The numbers indicated the distance to transcription start sites (TSS) of the corresponding gene. Data are presented as the mean ± SD. *$P < 0.05$.
